# Enhancing veteran care through relational facilitation: strategies and mixed methods outcomes

**DOI:** 10.1186/s43058-026-01065-z

**Published:** 2026-08-06

**Authors:** Heidi Sjoberg, Lauren McKown, Marina McCreight, Gretchen C. Stage, Summer Huang, Marcie Lee, Rachael Kenney, Brianne Morgan, Reed Hale, Joseph Frank, Christine D. Jones, Heather M. Gilmartin

**Affiliations:** 1 Denver/Seattle Center of Innovation for Veteran-Centered and Value Driven Care, VA Eastern Colorado Health Care System, 1700 N Wheeling St, Aurora, CO A2-329A USA; 2 University of Colorado, Anschutz Medical Campus, 13001 E 17th Pl, Aurora, CO 80045 USA; 3 Institute for Health Research, Kaiser Permanente Colorado, 2550 S Parker Rd, Aurora, CO 80014 USA

**Keywords:** Relational facilitation, Relational coordination, Implementation facilitation, Implementation strategy, Veterans health administration

## Abstract

**Background:**

Veterans receiving care in Veterans Health Administration (VA) and non-VA settings often face fragmented care coordination, leading to adverse outcomes. Our study aimed to enhance Veteran care coordination through three evidence-based interventions. Relational Coordination (RC) theory emphasizes enhancing communication and relationships to strengthen team dynamics, which improves care quality. This study evaluated whether RC guided facilitation (relational facilitation) enhanced collaboration and coordinated care within and between teams during intervention implementation.

**Methods:**

From 2021 to 2025 trained facilitators supported intervention implementation across 15 VAs using relational facilitation. Key activities included team role identification, relational map development, RC Survey deployment, and RC-related SMART (specific, measurable, attainable, relevant, time-based) goal setting. Relational facilitation impact was assessed using mixed methods incorporating survey data and participant interviews.

**Results:**

Nine sites (*n* = 138/1017 participants) completed pre/post surveys, with 8.3% average increase post-implementation for most sites (*n* = 7/9). Participants reported relational facilitation strengthened team dynamics and improved care coordination.

**Conclusion:**

Relational facilitation enhanced teams’ collaboration and intervention implementation. Relational facilitation can improve collaboration and patient outcomes.

**Supplementary Information:**

The online version contains supplementary material available at https://doi.org/10.1186/s43058-026-01065-z.

## Contributions to the literature

- This manuscript presents details on how to utilize an effective, novel implementation strategy – relational facilitation.
- This study contributes to the growing body of literature on RC and implementation facilitation by offering a replicable model for implementation and highlighting the resilience of clinical teams when equipped with the right tools and support.
- This manuscript addresses the lack of mixed methods data reported on Relational Coordination and changes in Relational Coordination over time by presenting a longitudinal, multi-site evaluation of Relational Coordination within and between clinical teams, using both quantitative and qualitative data.

## Background

Delivering high-quality, safe healthcare to Veterans is inherently complex due to their frequent use of both Veterans Health Administration (VA) and community healthcare services. The VA Maintaining Internal Systems and Strengthening Integrated Outside Networks (MISSION) Act expanded access through the Community Care Program, increasing dual-system utilization [1, 2]. While this expansion improves access to care and services without preauthorization, it also heightens the risk of fragmented and poorly coordinated care, resulting in adverse health outcomes, negative patient experiences, and increased costs [3, 4]. Although the VA has developed evidence-based practices to address care coordination challenges, their implementation remains difficult, particularly for Veterans with complex needs [5]. On average, it takes 17 years to implement only14% of evidence-based interventions into routine practice [6–8]. However, targeted implementation strategies, methods used to promote evidence-based intervention implementation, adoption, and integration, can accelerate this process [9–11].

One such strategy is relational facilitation, a six-step approach developed and piloted from 2018 to 2020 for the VA Triple Aim Quality Enhancement Research Initiative (QUERI) and the VA Office of Rural Health Transition’s Nurse Program [12]. Designed to support busy clinical teams, evaluators, and research or quality improvement studies, relational facilitation promotes adoption and sustainment of evidence-based interventions by strengthening communication and relationships within and between interdisciplinary teams. Relational facilitation addresses common barriers to implementation by enhancing teamwork, expanding social networks, and fostering collaboration [9–11].

Relational facilitation integrates two foundational strategies: Relational Coordination (RC) [13–20] and implementation facilitation [21]. RC theory emphasizes frequent, timely, accurate, and problem-solving communication, alongside shared goals, shared knowledge, and mutual respect [13–20]. These elements are key for effective task integration and team dynamics. Implementation facilitation supports adoption through active learning, participatory decision-making, and collaborative problem-solving and is led by internal or external facilitators [21]. Despite the proven value of facilitation in healthcare settings, relational aspects such as communication and team relationships are often overlooked due to demanding clinical workloads [22–24]. Relational facilitation addresses this gap by combining RC principles with traditional facilitation methods to improve implementation outcomes through stronger team engagement and coordination. The relational facilitation process is outlined and publicly available in the Relational Facilitation Guidebook. The Guidebook was finalized in February 2023 to make concepts of RC more accessible to VA researchers, evaluators, and clinicians.

From 2020 to 2025, the VA Office of Research and Development funded the Quadruple Aim QUERI (QA-QUERI) to improve care coordination for Veterans. QA-QUERI supported the implementation of three evidence-based care coordination interventions implemented across geographically diverse VA sites throughout the United States, serving Veterans in urban, rural, and highly rural areas. These interventions were: 1) Care Coordination and Integrated Case Management (CCICM), a VA nationally mandated practice change that assigned a nurse or social worker to provide intensive case management and care coordination to high risk Veterans with moderate to complex care needs [1, 25, 26]; 2) the rural Transitions Nurse Program with Home Health Care (TNP-HHC) intervention, which assigned a nurse or social worker to support Veterans with their transitions from VA hospitalization to home with home health services [12, 27]; and 3) Whole Health Coaches in Pain Management Teams (WHCPMT) intervention, which supported and promoted the integration of Whole Health Coaches into Pain Management Teams to enhance care for Veterans experiencing chronic pain. QA-QUERI intervention details are outlined in Table 1.

**Table 1:** The three evidence-based interventions, partners, start Year, and number of sites

Intervention	Evidence-Based Practices	Partners	Start Year	Number of Sites
Care Coordination and Integrated Case Management	Enrolled Veterans are assigned a Lead Coordinator clinician to be their primary contact, address medical, social, and behavioral health needs, and improve care coordination.	Executive sponsor, nurse and social worker co-coordinators, data analysts, group practice managers, information technology specialists, chief nurses, chief social workers, executive officers, nurse and social worker consultants from National Veterans Health Administration offices	2020	6
Transitions Nurse Program with Home Health Care	The nurse or social worker transitions coordinator collaborated with medical teams and worked with Veterans to address medical and social needs and improve care transitions.	Nurses, nurse managers, social workers, executive officers, associate directors, deputy associate directors, chief nurses, and chief social workers	2021	3
Whole Health Coaches in Pain Management Teams	The implementation team provided relational facilitation and education and offered individualized Whole Health Coach coaching support to improve care coordination.	Whole Health Coaches, doctors, psychologists, psychiatrists, social workers, nurses, medical support assistants, program managers, Pain Management, Opioid Safety, and Prescription Drug Monitoring Program coordinators	2023	6

Each site worked with the QA-QUERI team to implement one of these interventions. Interventions were rolled out from 2020 to 2025 following a stepped-wedge design [28]. Implementation of these interventions was led locally at each site by VA site teams, with support from the multidisciplinary QA-QUERI evaluation team. The QA-QUERI team included program managers, social workers, nurses, physicians, nurse scientists, implementation scientists, and experts in qualitative, quantitative, and RC methods. Together, the site teams and the QA-QUERI team collaborated to ensure effective implementation of the interventions tailored to the needs of each site. Relational facilitation supported implementation of QA-QUERI interventions.

The objective of this manuscript is to present the first longitudinal, multi-site study of relational facilitation, describing its impact on the implementation of QA-QUERI interventions. The Revised Standards for Quality Improvement Reporting Excellent (Additional file 1) [29] informed the writing of this manuscript.

## Methods

This convergent sequential mixed methods study aimed to understand the impact of relational facilitation on collaboration and coordinated care within and across teams who implemented one of the three QA-QUERI interventions.

### Relational facilitation

Four facilitators from the QA-QUERI study team were trained in RC theory and methods during a three-day workshop hosted by the Relational Coordination Collaborative. Following the workshop, facilitators participated in six months of coaching sessions with RC content experts to address implementation barriers and strengthen facilitators for RC adoption. During this period, the relational facilitation strategy was field tested and refined to improve its applicability in the QA-QUERI settings.

**Fig. 1:**
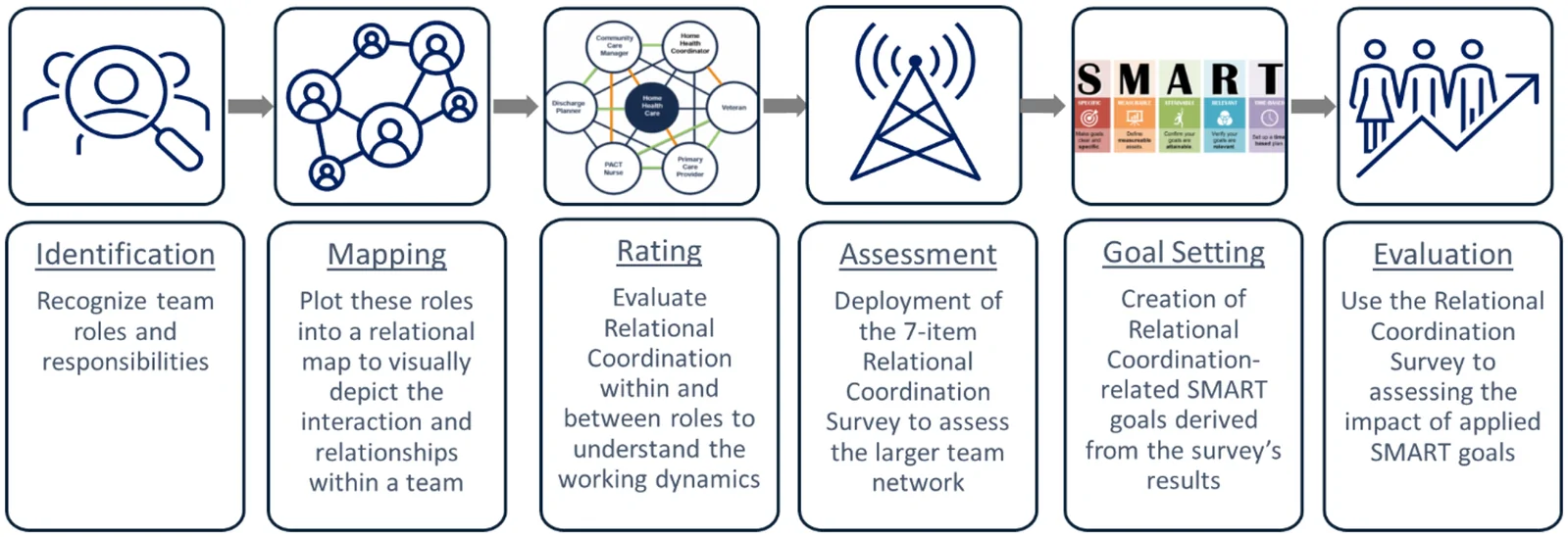
Relational facilitation six-step implementation process

Within QA-QUERI, relational facilitation was applied during both pre-implementation and implementation phases. Facilitators embedded assessment, goal setting, and team building activities into existing virtual meetings between the QA-QUERI study team and site staff to minimize disruption and enhance integration. Relational facilitation activities (Fig. 1) began with QA-QUERI sites in July 2021. The activities were conducted as part of a VA operational project and did not constitute research, in whole or in part, in accordance with VA Handbook 1058.05. Therefore, institutional review board approval was not required.

### Pre-implementation activities

**Fig. 2:**
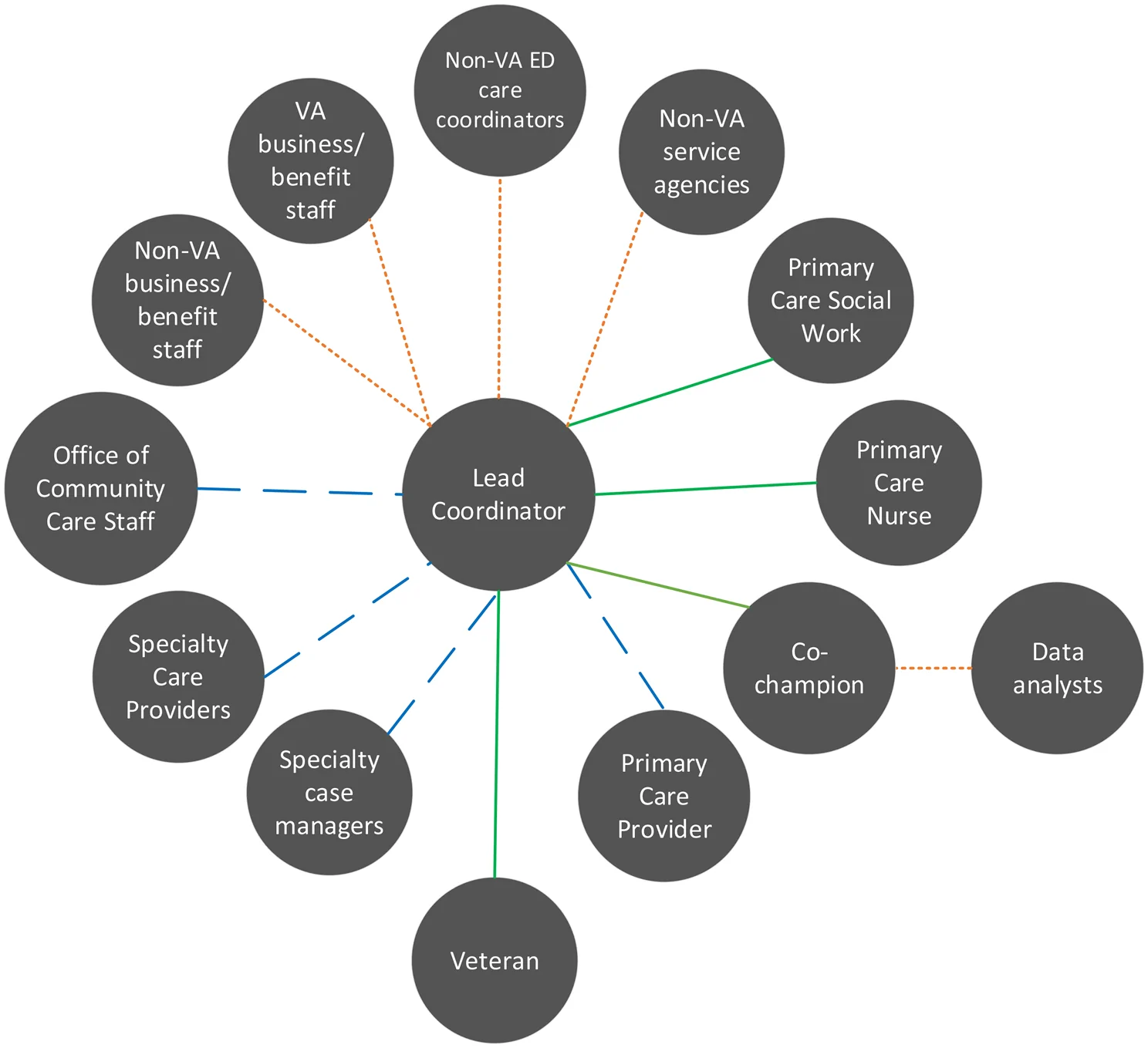
Example of a finalized relational map. Note. Abbreviations: Veterans Health Administration (VA); Emergency Department (ED)

QA-QUERI facilitators were part of the QA-QUERI study team that was based at a VA external to the 15 sites. Two QA-QUERI facilitators and four to 10 site participants attended each meeting. The facilitators began with standardized education on RC theory (~45–60 minutes) followed by guided relational mapping exercises. Participants at some sites completed the relational mapping exercises in one meeting (~60 minutes), whereas other sites completed the exercises over two meetings (~60 minutes each) as they had lengthier discussions around what roles should be on their relational map and the quality of RC between roles. Facilitators drafted initial maps outlining team roles, which site staff then customized to reflect their local teams. Participants rated the perceived quality of communication and relationships between roles on a 1–3 scale (1 = low, 2 = moderate, 3 = strong). Finalized relational maps (see Fig. 2 for an example) were shared with sites to reference throughout implementation.

### Relational coordination survey administration

Each site team aimed to improve care coordination between roles that were collaborating on one of the three interventions described previously (see Background section). During implementation, the RC Survey, a validated instrument measuring the seven RC dimensions, was used to assess team communication and relationships [17] and identify areas of opportunity that needed targeted interventions. Survey items were rated on a 1–5 Likert scale (Table 2). Participants evaluated relationships between roles rather than individuals to identify strong and weak ties. RC Analytics administered the survey via an online platform, using email addresses provided by site leads. Surveys were completed either during virtual meetings or during the workday. Surveys took on average 10 minutes to complete. The initial RC Survey was administered pre-SMART (specific, measurable, attainable, relevant, time-based) goal setting within three months of intervention launch and remained open for 10 days. One reminder email was sent out seven days after the initial survey email was sent. Consent for survey participation was implied by participants upon submission of the RC Survey. The RC Survey was distributed to staff across roles and disciplines at each site to capture multiple perspectives and enhance response rates. RC Analytics generated standardized reports summarizing site and role-level scores, including a composite RC Index. Data were deidentified, aggregated, and analyzed descriptively to identify trends across the seven RC dimensions.

**Table 2:** Relational coordination survey questions

Relational Coordination Dimension	Definition	Survey Question	Response Options
Frequent Communication	Degree in which people communicate with each other about their work.	How frequently do people in these groups communicate with you about [work process]?	Not nearly enough (1), Not enough (2), Just the right amount (3), Too often (4), Much too often (5)
Timely Communication	How quickly people report important details about a work process.	How timely is their communication with you about [work process]?	Never (1), Rarely (2), Sometimes (3), Often (4), Always (5)
Accurate Communication	Exactness of details relayed between people about a work process.	How accurate is their communication with you about [work process]?	Never (1), Rarely (2), Sometimes (3), Often (4), Always (5)
Problem-solving Communication	Degree in which people look for resolutions instead of blaming others when problems arise.	When there is a problem in [work process], do people in these groups blame others or try to solve the problem?	Always blame (1), Mostly blame (2), Neither blame nor solve (3), Mostly solve (4), Always solve (5)
Shared Goals	Comprehension of how each other’s work supports each other in the same work process.	How much do people in these groups share your goals in [work process]?	Not at all (1), A little (2), Somewhat (3), A lot (4), Completely (5)
Shared Knowledge	Awareness of other’s roles and responsibilities and who needs to know what by when.	How much do people in these groups know about the work you do with [work process]?	Nothing (1), A little (2), Some (3), A lot (4), Everything (5)
Mutual Respect	Appreciating other’s work.	How much do people in these groups respect the work you do with [work process]?	Not at all (1), A little (2), Somewhat (3), A lot (4), Completely (5)

### Goal development

Survey results were presented by QA-QUERI facilitators to site teams during virtual meetings (~30 minutes) and shared with additional partners via email (see Additional file 2 for an example presentation). Teams identified their strengths and gaps in communication and relationships, then developed SMART goals (~10 minutes) to address areas for improvement. Common goals that site teams developed included hosting monthly lunch-and-learns, initiating internal communities of practice, and conducting educational outreach to other programs during daily huddles. Progress was monitored in ongoing meetings, and new goals were developed as previous ones were completed.

### Post-implementation assessment

A post-SMART goal implementation RC Survey was administered six to nine months after initial SMART goal development. The RC Survey took 10 minutes on average to complete. RC Analytics provided deidentified comparison reports of pre- and post-survey data. Facilitators presented these results to site teams in existing virtual meetings (~30 minutes), marking the end of active relational facilitation.

### Quantitative analysis

Due to limited response rates, statistical analyses was constrained. Surveys were independent, meaning different individuals may have completed the pre- and post-surveys. As a result, we relied on descriptive statistics and trend analysis, without accounting for within-subject or site-level correlations. A large sample size would be required for robust statistical modeling.

### Qualitative data collection

Following the post-survey, the QA-QUERI qualitative team conducted interviews (and multiple focus groups for the QA-QUERI CCICM intervention) with key staff from each intervention site. Interview guides (Additional file 3) focused on the perceived impact of relational facilitation on care coordination and team collaboration. The qualitative team used purposive sampling wherein participants were selected based on their involvement in relational facilitation activities, intervention implementation, and availability for an in-depth discussion [30]. Interview participants were recruited from staff who were invited to complete the RC Survey since interview questions were about RC and relational facilitation. However, interview participants may not have also completed the RC Survey since the results were de-identified. Interviews and focus groups averaged one hour, were recorded, transcribed verbatim, and stored securely behind the VA firewall.

### Qualitative analysis

Data were analyzed deductively using a priori categories informed by the RC dimensions. Then these a priori categories were cross-referenced with the results from the quantitative data to determine any emergent categories [31]. Data was managed in Atlas.ti 7. Two interviews per intervention (*n* = 6) were double coded by qualitative team members, followed by weekly consensus meetings to resolve discrepancies. Remaining interviews were coded and summarized collaboratively (MM, GS, ML, LM). Emergent themes were integrated into quantitative findings to enrich interpretation.

### Mixed methods analysis

**Fig. 3:**
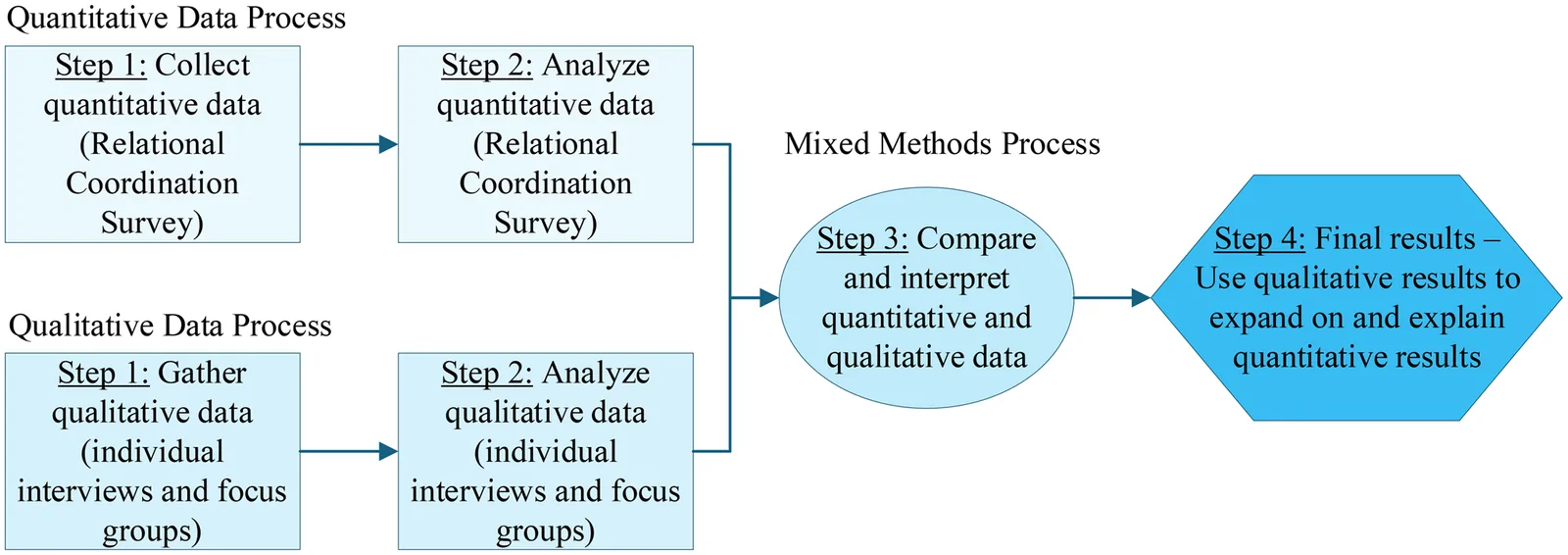
Convergent sequential mixed methods (QUAN→ qual) design

The QA-QUERI study team employed a convergent sequential mixed methods design (QUAN ${\bf{\rightarrow}}$ qual) [32, 33] to expand on the quantitative results with contextual narratives to evaluate delivery and impact of the relational facilitation strategy. A QUAN ${\bf{\rightarrow}}$ qual convergent sequential mixed methods design was used to explain not just what happened, but how and why. Quantitative and qualitative data were collected across QA-QUERI sites, allowing for intra-method data integration (Fig. 3).

First, the study team administered the RC Survey to obtain and analyze quantitative data from clinicians and staff involved with intervention implementation and delivery or related care processes. Next, the qualitative team conducted individual interviews (and focus groups for CCICM sites) with key participants from each intervention at each VA site. Interview guides included questions targeted towards understanding the impact of relational facilitation activities. Qualitative findings were used to expand on the quantitative results and provide more depth to further assess and understand changes between pre- and post-SMART goal setting RC Surveys at each site with each intervention. The QA-QUERI study team used an independent analytical approach [32] – quantitative and qualitative data were collected and analyzed separately and then integrated (Fig. 3). The integration intent was to expand understanding between the quantitative and qualitative data sets to allow for a more comprehensive and rich interpretation of the RC Survey results. For each RC construct, the QA-QUERI study team assessed where quantitative data converged and diverged with qualitative data.

## Results

All 15 QA-QUERI sites engaged in at least some of the relational facilitation activities, including RC education, relational mapping, and completion of the RC Survey before SMART goal setting. Nine of the 15 sites proceeded to develop and implement RC-related SMART goals and completed the post-SMART goal completion RC Survey. Six sites opted out of completing the SMART goal development and the post-RC Survey due to competing priorities, limited staff capacity, or implementation support ended at their site.

### Relational coordination survey and interview completion

A total of 590 staff across sites and interventions were invited to complete the pre-SMART goal setting RC Survey, with 191 completing the survey (average completion rate: 43.3%; range: 5–30 responses per site; Standard Deviation [SD]: 26.60). For the post-SMART goal setting RC Survey, 1,017 staff were invited and 138 completed the survey (average completion rate: 23.10%; range: 5–32 responses per site; SD: 14.80). The lower average completion rates of the post-SMART goal setting RC Survey was largely due to a broader invitation pool, with two sites inviting 6–10 times more participants than in the pre-SMART goal setting RC 

Survey. Table 3 details participant distribution by site.

**Table 3:** Pre- and post- relational coordination survey responses

Site	Survey	Invited	Completed	Response Rate
Veterans Health Administration (VA) 1	Pre Survey	25	19	76%
	Post Survey	183	32	17%
VA 2	Pre Survey	37	9	24%
	Post Survey	442	30	7%
VA 3	Pre Survey	54	14	26%
	Post Survey	48	7	15%
VA 4	Pre Survey	43	9	21%
	Post Survey	48	13	27%
VA 5	Pre Survey	136	23	17%
	Post Survey	161	18	11%
VA 6	Pre Survey	31	6	19%
	Post Survey	35	5	14%
VA 7	Pre Survey	62	15	24%
	Post Survey	61	16	26%
VA 8	Pre Survey	46	10	22%
VA 9	Pre Survey	31	5	16%
VA 10	Pre Survey	12	7	58%
VA 11	Pre Survey	25	13	52%
VA 12	Pre Survey	26	24	92%
	Post Survey	22	7	33%
VA 13	Pre Survey	29	12	41%
VA 14	Pre Survey	8	7	88%
	Post Survey	17	10	59%
VA 15	Pre Survey	25	18	72%
Total Pre Survey		590	191	32%
Total Post Survey		1017	138	14%

*Note.* Abbreviations: Veterans Health Administration (VA)

In total, 105 interviews and focus groups were completed: 98 individual interviews across 13 sites and 7 focus groups at six sites. Table 4 provides a breakdown by site.

**Table 4:** Interview and focus group counts by site

Site	Roles	Individual Interviews	Focus Groups (number of participants)	Total Participants
Veterans Health Administration (VA) 1	Nurses, social workers, managers, clinical leaders	13	1 (8 participants)	21
VA 2	Nurses, social workers, managers, clinical leaders	8	1 (14 participants)	22
VA 3	Nurses, social workers, managers, clinical leaders	10	2 (10 participants)	20
VA 4	Nurses, social workers, managers, clinical leaders	10	1 (14 participants)	24
VA 5	Nurses, social workers, managers, clinical leaders	15	1 (4 participants)	19
VA 6	Nurses, social workers, managers, clinical leaders	14	1 (6 participants)	20
VA 7	Nurses, social workers, managers, clinical leaders	2	0	2
VA 8	Nurses, social workers, managers, clinical leaders	2	0	2
VA 9	Nurses, social workers, managers, clinical leaders	1	0	1
VA 10	None	0	0	0
VA 11	None	0	0	0
VA 12	Whole Health Coaches, Pain Management Team clinicians	8	0	8
VA 13	Whole Health Coaches, Pain Management Team clinicians	4	0	4
VA 14	Whole Health Coaches, Pain Management Team clinicians	5	0	5
VA 15	Whole Health Coaches, Pain Management Team clinicians	6	0	6
Total Participants		98	56	154

*Legend.* VA 10 and VA 11 did not finish all relational facilitation activities and dropped out of the intervention early. Thus, they did not complete any interviews or focus groups

*Note. *Abbreviations: Veterans Health Administration (VA)

### Relational coordination index scores: pre- and post-implementation

**Fig. 4:**
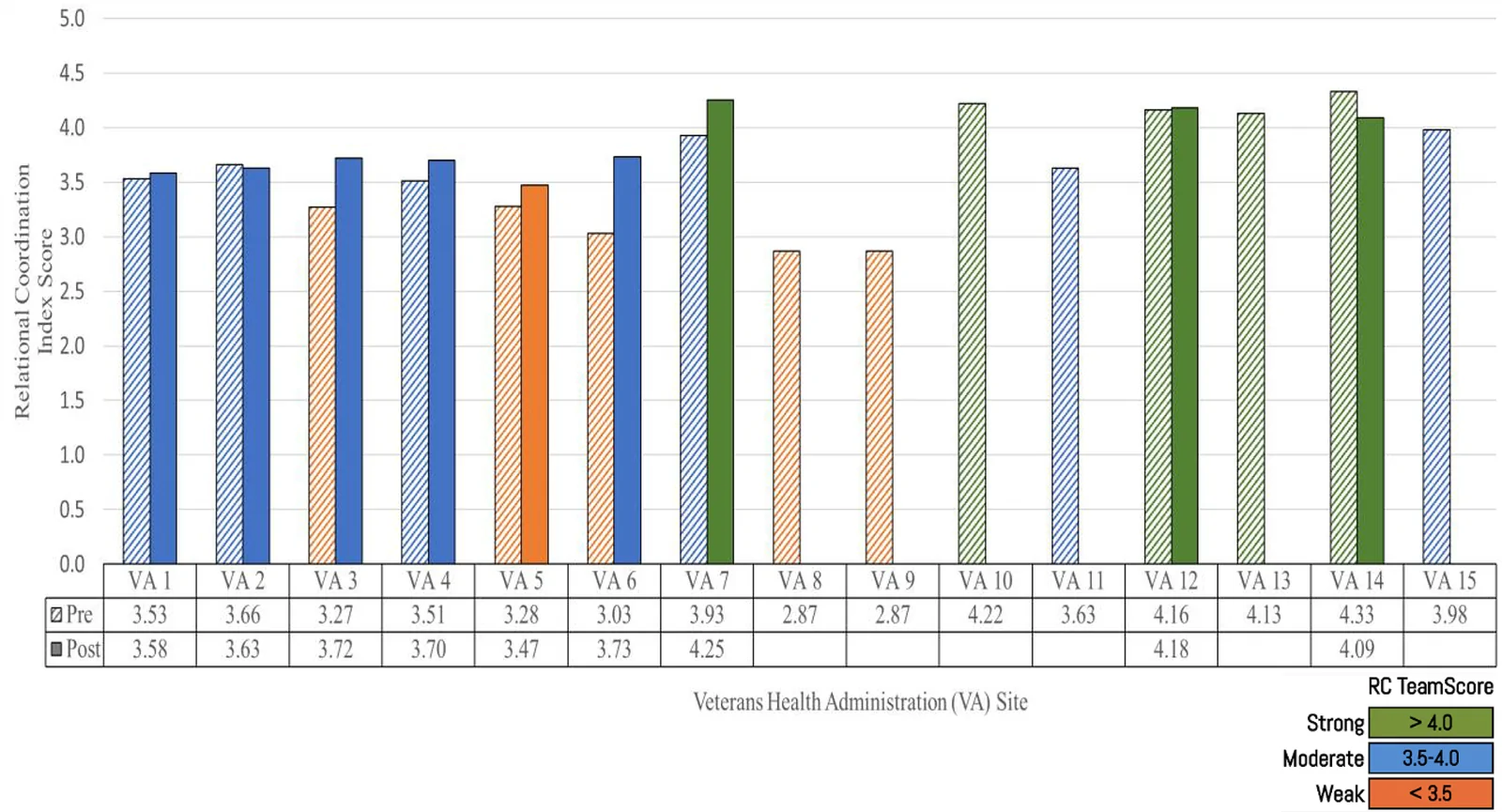
Pre- and post-implementation relational coordination survey scores Note. Abbreviations: Veterans Health Administration (VA)

Pre-implementation RC Index scores across all 15 sites ranged from 2.90 (weak) to 4.30 (strong) with an average score of 3.63 (SD: 0.47) (moderate) across sites. Among the nine sites that completed post-SMART goal setting RC Surveys, scores ranged from 3.50 (weak) to 4.30 (strong) with an average score of 3.81 (SD: 0.27) (moderate). Seven of these sites showed improvement, with an average increase of 8.30% (range: 0.48% − 23.10%; SD: 7.90). Two sites experienced a decline, averaging a 3.20% decrease (range: 0.82% − 5.50%; SD: 3.30). Figure 4 illustrates site-level changes in RC Index scores.

### Changes in Relational Coordination Survey dimensions

**Fig. 5:**
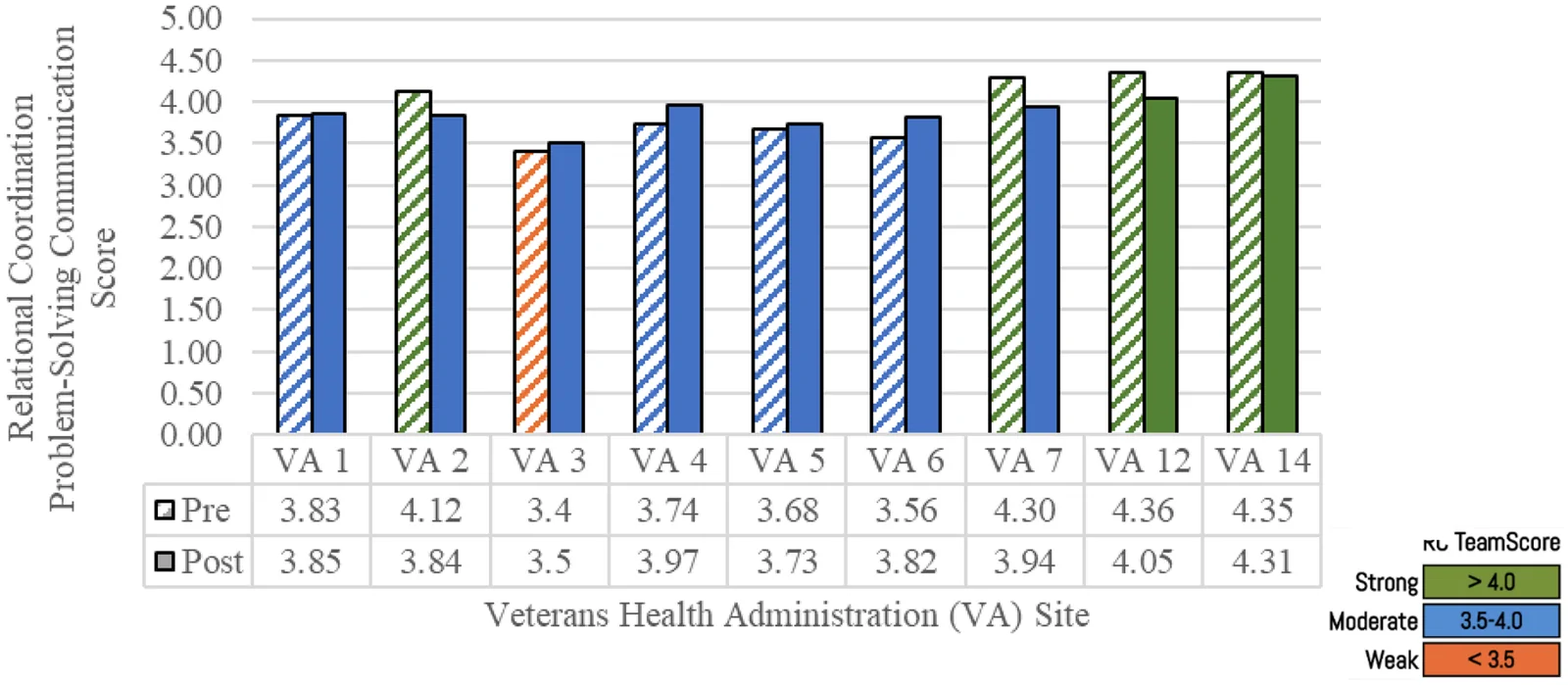
Problem-solving communication improved for most sites. Note. Abbreviations: Veterans Health Administration (VA)

**Fig. 6:**
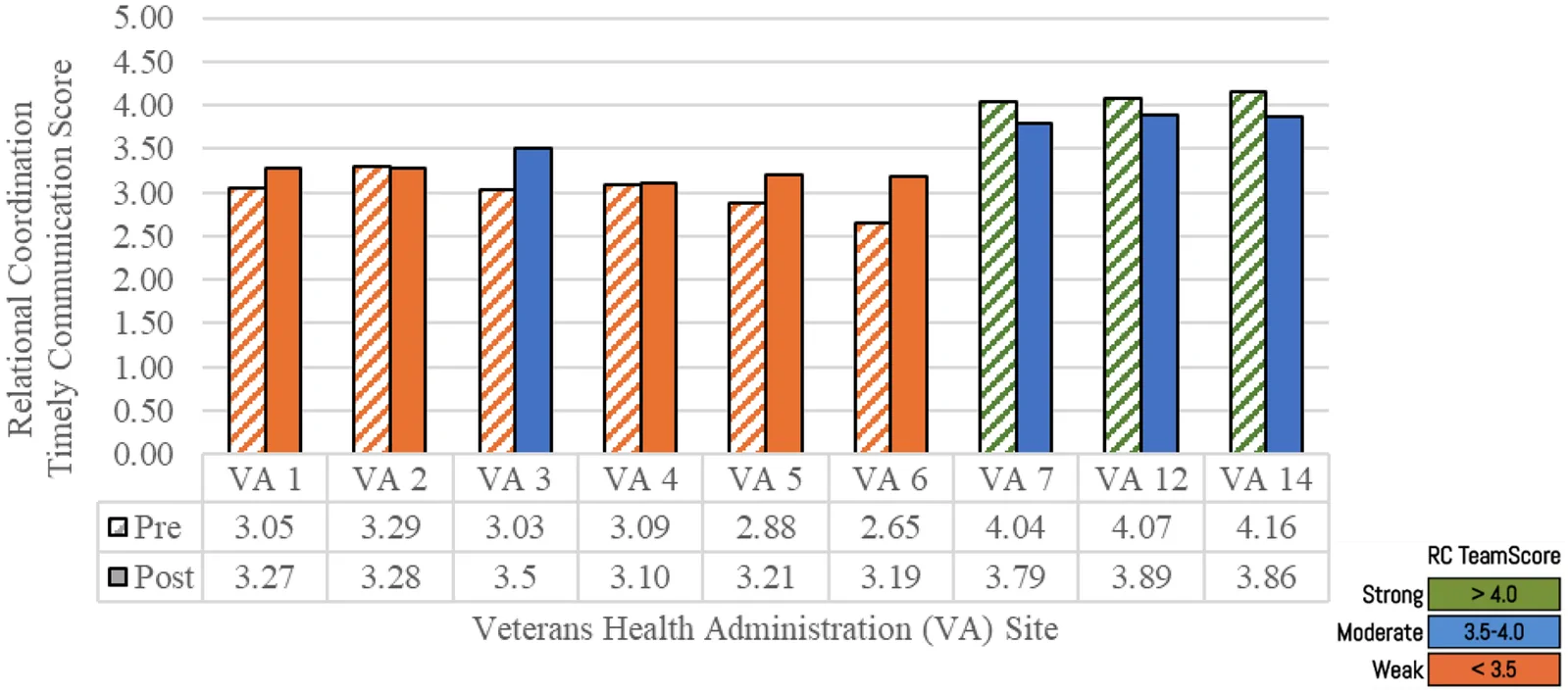
Timely communication improved for five out of nine sites. Note. Abbreviations: Veterans Health Administration (VA)

**Fig. 7:**
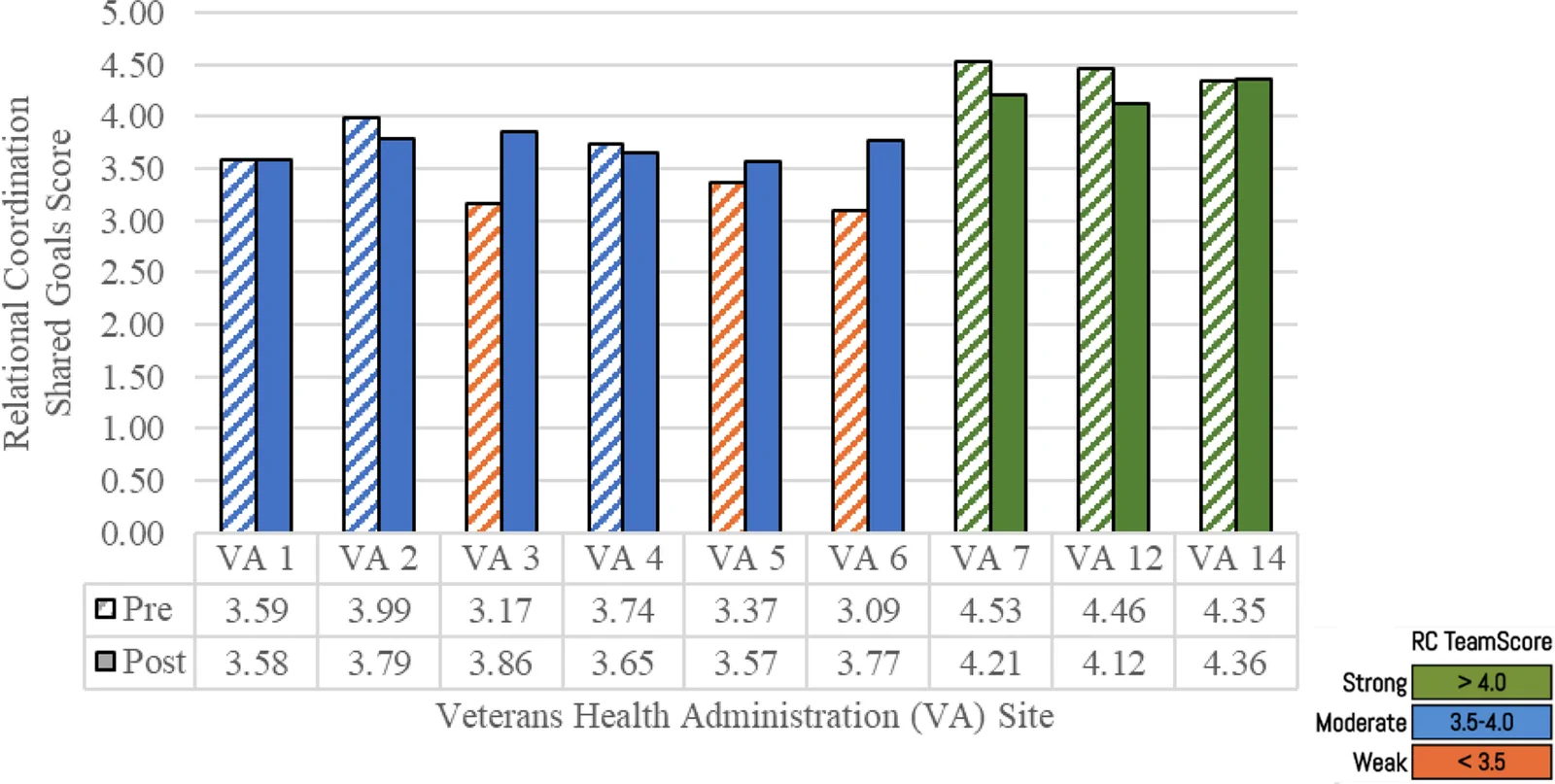
Shared goals changed inconsistently between sites. Note. Abbreviations: Veterans Health Administration (VA)

**Fig. 8:**
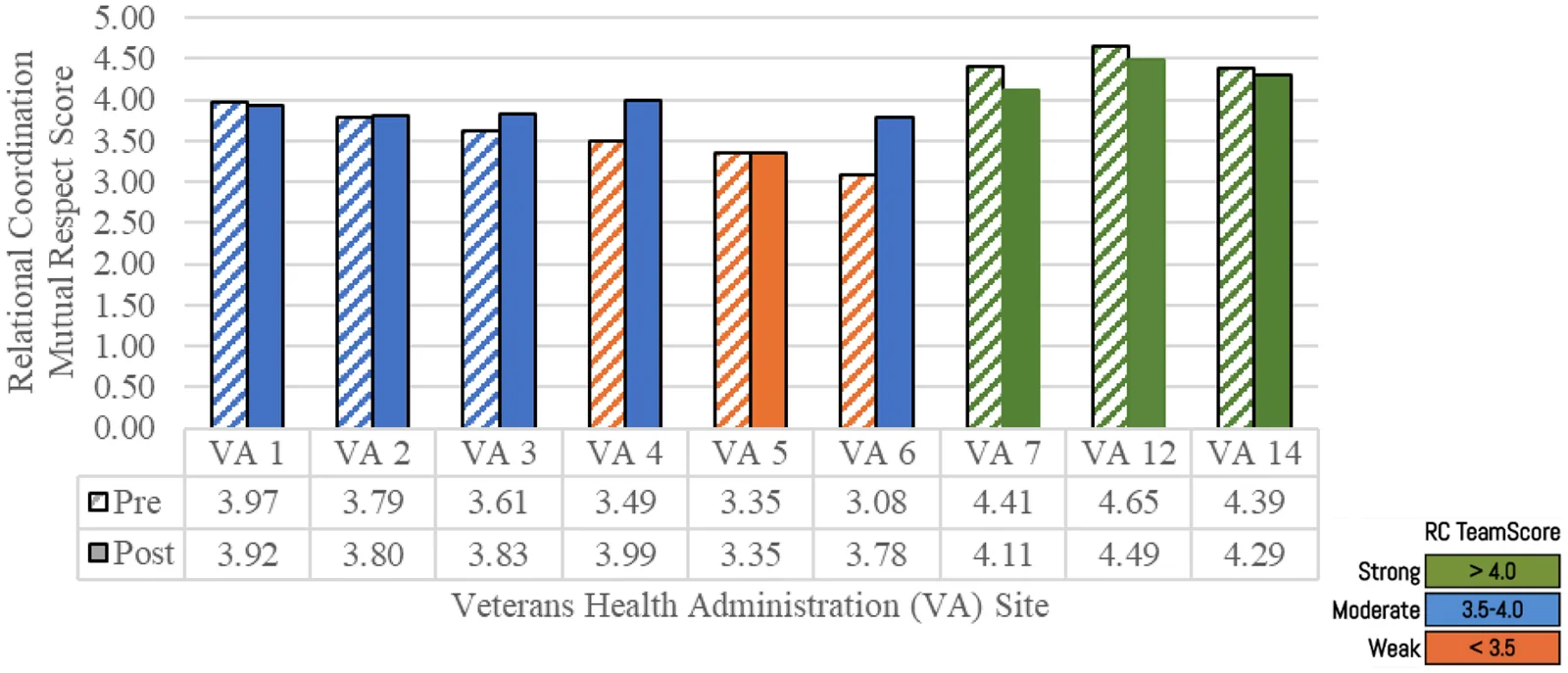
Mutual respect inconsistently improved, decreased, or remained the same across sites. Note. Abbreviations: Veterans Health Administration (VA)

**Fig. 9:**
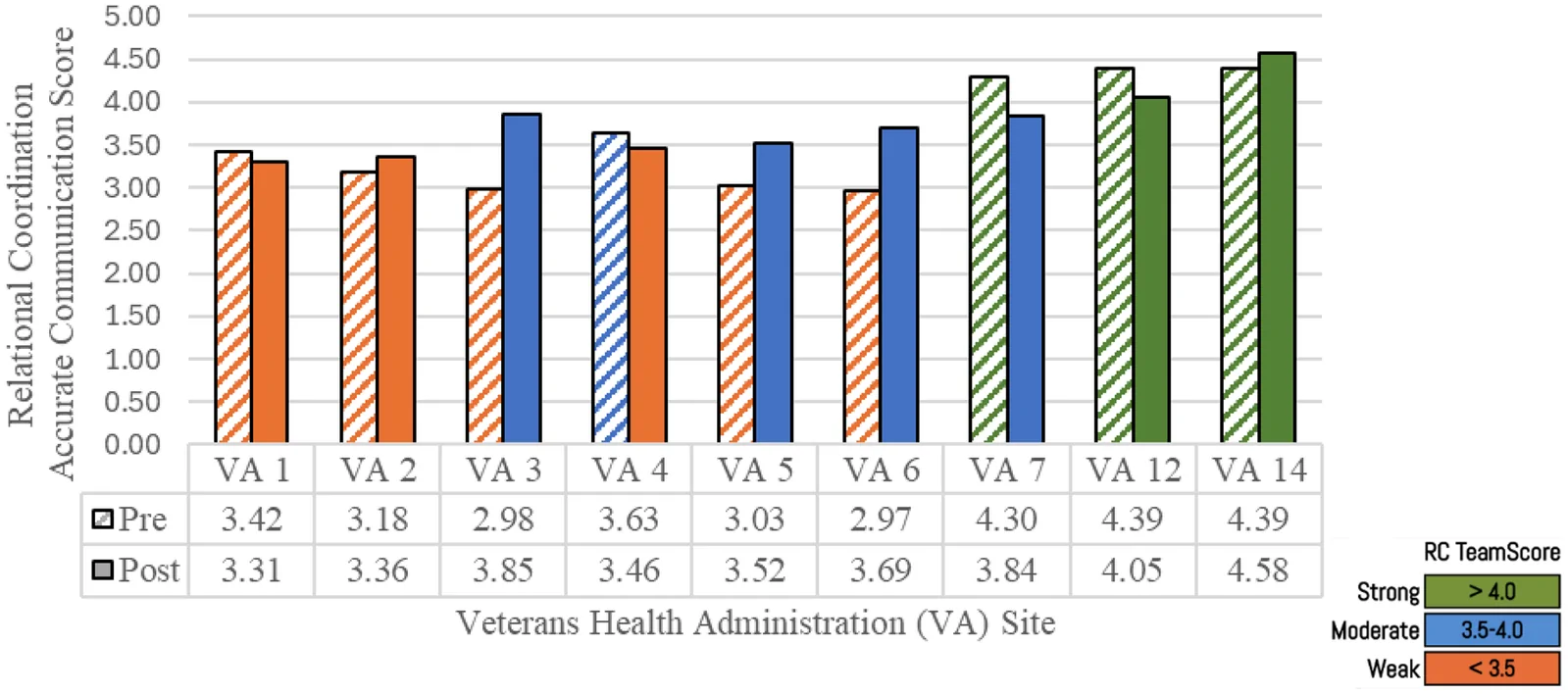
Accurate communication improved for five of nine sites. Note. Abbreviations: Veterans Health Administration (VA)

**Fig. 10:**
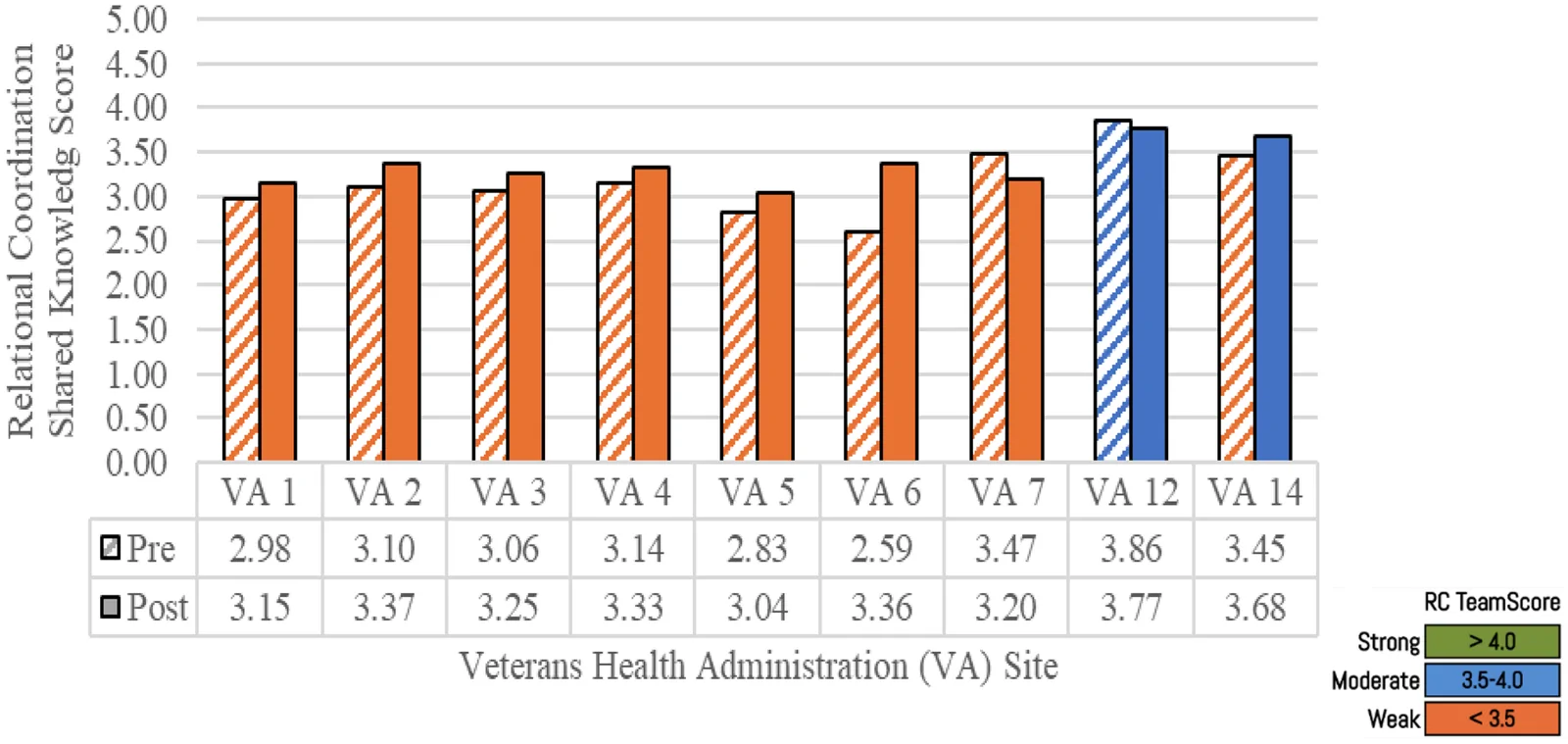
Shared knowledge had the lowest scores across sites from pre- to post- surveys. Note. Abbreviations: Veterans Health Administration (VA)

**Fig. 11:**
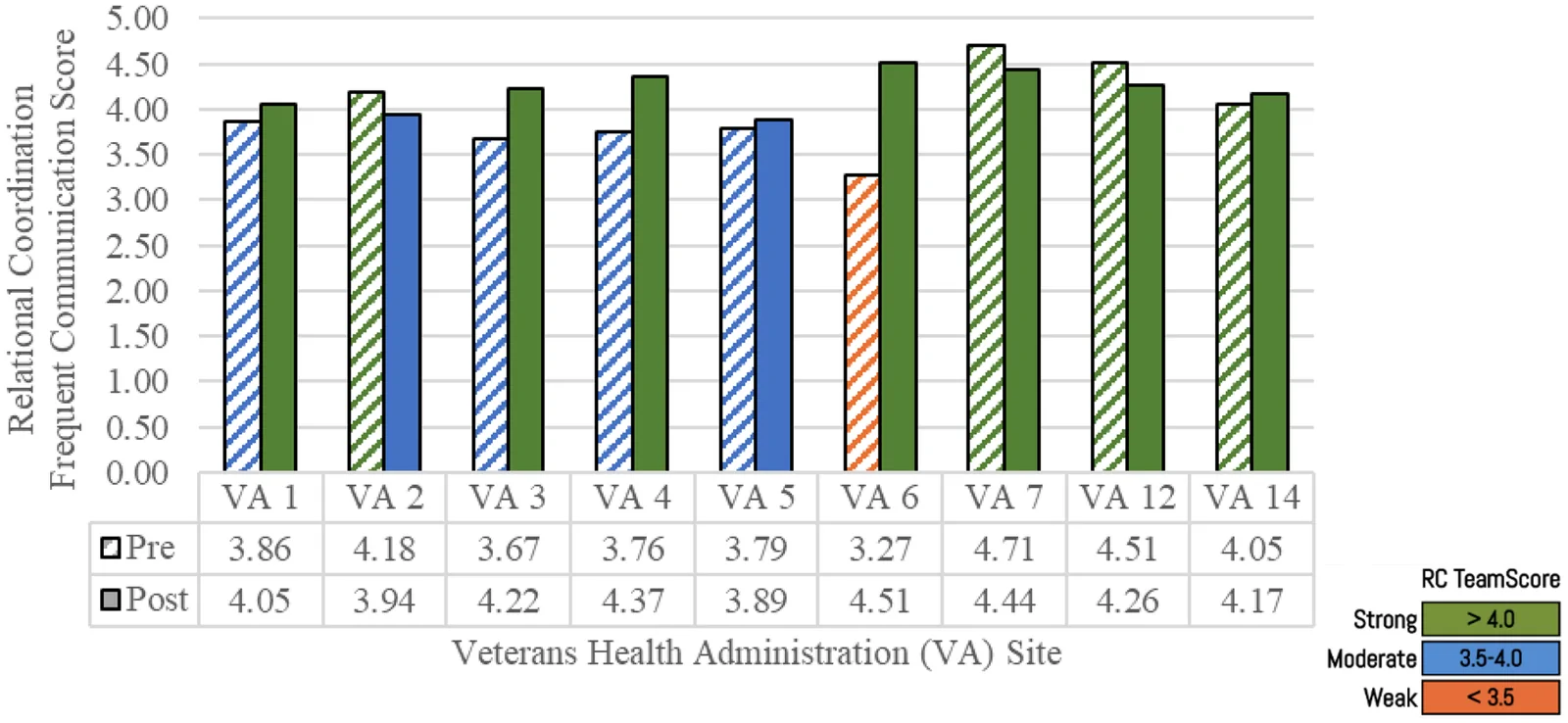
Frequent communication increased for most sites. Note. Abbreviations: Veterans Health Administration (VA)

Across the nine sites with pre-post RC Survey data, most RC dimensions improved following relational facilitation and SMART goal completion. RC dimension average change across sites are reported below in ascending order starting with the RC dimension that experienced the least average change from pre-post surveys. Problem-solving communication remained relatively stable, moving from an average of 3.93 (SD: 0.34) to 3.89 (SD: 0.21). Timely communication rose slightly on average from 3.40 (SD: 0.40) to 3.45 (SD: 0.30). Shared goals improved on average from 3.81 (SD: 0.52) to 3.88 (S: 0.27). Mutual respect increased on average from 3.86 (SD: 0.51) to 3.95 (SD: 0.31), with one site showing no change. Accurate communication increased on average from 3.60 (SD: 0.58) to 3.74 (SD: 0.38). Shared knowledge, the lowest-scoring dimension, rose from an average of 3.16 (SD: 0.36) to 3.37 (SD: 0.23). The nine sites that developed SMART goals specifically targeted the shared knowledge dimension. Frequent communication improved from an average of 3.98 (SD: 0.42) to 4.20 (SD: 0.20). Figures 5, 6, 7, 8, 9, 10, 11 display absolute changes for each site’s unique RC dimensions.

### Qualitative results

Interviews were completed at six CCICM sites, three TNP-HHC sites, and four WHCPMT sites. In addition to interviews, focus groups were conducted at all CCICM sites. Table 4 provides complete counts of interviews and focus groups. To protect the confidentiality of participants, specific roles are not detailed; however, most participants across the three interventions were clinicians, including social workers and registered nurses, and administrative staff that were tasked with supporting, implementing, and delivering one of the three interventions.

Qualitative results provided insight into how the quality of RC changed within and between teams over time. RC communication dimensions improved since staff at sites were motivated to streamline communication processes and enhance Veteran care. Frequent communication increased when other staff understood roles and responsibilities of those implementing the intervention:

*… people come to us ‘cause they realize what we do now, it’s good - we are that middle person. So, I feel like our communication has definitely increased and improved.* Clinician 81

Clinicians noted that with instances where timely communication improved, Veterans received better care:

*I think the benefits are for the Veteran, that they are getting their needs met more efficiently…* Clinician 12

However, timely communication did not always occur, which added to clinicians' workload:

*I had to catch the patient before they were discharged and since I didn’t have anyone on the inpatient side to tell me really when the patient was going home, like we looked at, OK, can we go to discharge planning rounds, but those were long. Those were like hours and hours … I’m doing my other role, my full-time job, and then to attend discharge rounds, that wasn’t really that helpful.* Clinician 20

At sites where accurate communication occurred, collaboration between disciplines improved:

*I think it’s a true multidisciplinary practice … rather than all the specialties kind of working in parallel, everybody really synchronizes and sort of provides their expertise in a real multimodal way … it’s just a very collegial environment, you know, with really excellent collaboration.* Clinician 23

Many clinicians were dedicated to successfully implementing the QA-QUERI intervention at their site by increasing problem-solving communication:

*I would say helping Veterans out, identifying problems before they turn into bigger issues, and being part of a process to fix problems.* Clinician 81

RC relational dimensions also changed across sites throughout implementation of the QA-QUERI interventions. Across interventions, many clinicians experienced improvements in shared goals within their teams:

*… everyone on this team really gave 100 percent every time, you know, really working because we really believe in what we do.* Clinician 43

At sites where staff lacked clarity on the intervention, shared goals between teams was lacking:

*There is a lack of unified purpose and understanding of the intervention at the site level … many frontline staff were unclear on the bigger educational plan and the intervention process at their sites.* Clinician 25

Staff reported being motivated to build relationships, understand other disciplines’ roles, and proactively share knowledge within and between teams to improve Veterans’ care:

*… relational coordination was a good reminder to not be afraid to ask questions and have meetings and make connections with people who impact the Veterans’ care and to be proactive about building those relationships and maintaining them.* Clinician 64

*… relational aspect - any time you run into an issue with a Veteran and care, make sure that you are handing it to the correct person and building relationships to the people who fix the, whatever problem it is, and over time, you will become more knowledgeable about how to approach issues and just problem-solving in general …* Clinician 65

*Well, that, yeah, we kind of developed, that’s when we developed our orientation actually so that we could invite other primary care providers or other specialty clinics or whatever to jump on the orientation so they could get an overview of [the] team.* Clinician 86

Lastly, qualitative results suggested that mutual respect generally strengthened across sites as the QA-QUERI interventions were implemented:

*And you have this group of peers from all different disciplines who are saying, yeah, you know, you’re right. And it’s validating. I think it’s really validating that you – you’re being – your work is being given credit and value, even when it feels like maybe you’re not necessarily reaching a Veteran. But someone else knows you’ve tried, I guess, because there’s a lot of stuff that we do every day that, you know, we’re not going to email our supervisor a synopsis of our day every day.* Clinician 85

Additional qualitative findings are detailed in the mixed methods section since they were used to expand on the quantitative results and provide a richer understanding of the impact of relational facilitation.

### Qualitative insights expanding on quantitative data

Qualitative data provided context to the quantitative data, revealing that improvements were not uniform across sites. For example, accurate communication showed the greatest average improvement (33.47%, *n* = 6), yet some sites reported declines due to organizational uncertainty stemming from the COVID-19 pandemic and VA policy changes across the multi-year implementation. One clinician noted:

*There’s all kinds of changes going on. So, a lot of our timelines have been either completely postponed or completely deleted because we’re still trying to figure out what in heaven’s name is going on with VA.* Clinician 63

Concerns about program sustainment also affected staff morale:

*And when all these cuts were coming at the federal level with our new administration … the number one question I got … is they’re not going to drop this program, are they? It’s been helpful and our Whole Health coach is amazing, and we were all worried too that the position would be dropped.* Clinician 38

Despite these challenges, most sites experienced improvements across RC dimensions.

Problem solving communication, while the least improved domain, still showed an average increase of 7.19% across eight sites. Qualitative data provided valuable context for the observed increase. Despite a challenging environment marked by hiring freezes and limited funding, clinicians demonstrated resilience and adaptability, often devising creative solutions to maintain care quality for Veterans. This resourcefulness was echoed in participant interviews:

*So, then you just have to make what you have work. And that’s kind of where we’ve always been successful in our strength. So, you kind of do what we got to do with what we have and make it work. So having that can-do attitude.* Clinician 11

However, this “can-do attitude” had its limits. In some cases, staff expressed frustration with being asked to do more with fewer resources, highlighting the emotional toll of sustained under-resourcing:

*There’s a lot of changes going on throughout the VA right now as well, a lot of staff shortages, people cannot give more than they’re already giving. … Every single person is being asked to do more with less, so people don’t really feel like they have the capacity for more right now. And again, you have to have the can-do attitude. I speak negatively because that’s my – that’s my venting process. So I do vent a lot because it frustrates me.* Clinician 11

Clinicians also described frequent team-based problem solving to address challenges in care coordination, such as navigating medication management across departments:

*I think one problem that we’re facing right now is pushback from primary care in resuming or continuing medication changes that has happened from our team … And so we discuss this during our team’s meetings … that we have four times a week. And then we come up with how we’re going to respond … I would say we talk about it and then decide how to respond as a team.* Clinician 86

These narratives underscore how behavioral dimensions, like problem-solving communication, influence clinician well-being and job satisfaction. Table 5 details the convergence and divergence of qualitative and quantitative findings across RC dimensions.

**Table 5:** Qualitative findings that explain Relational Coordination dimension changes

Relational Coordination Dimensions	Average Dimension Changes Across Interventions	Qualitative Data Across Interventions
Frequent Communication	Increase: 12.41% (*n* = 7) Decrease: 4.31% (*n* = 2)	At most sites, communication frequency increased. Factors that negatively impacted communication frequency included limited time and siloed departments. Convergent: “Communication, communication, communication. It’s really about understanding what [the intervention] is and does and your responsibility, your role, how it differentiates from your other roles.” (Clinician 4) Divergent: “I just don’t have capacity to do it … We touch base, here and there once or twice a week, and we brainstorm, and we come up with how that looks like, but it’s a lot of behind-the-scenes trying to get people to put presentations together, chasing people a lot to get the work, the stuff back, sending a lot of emails, updating notes, doing chart reviews. It’s a lot.” (Clinician 9)
Timely Communication	Increase: 10.82% (*n* = 8) Decrease: 0.3% (*n* = 1)	Timely communication increased at most sites; however, staff reported it was difficult to sustain timely communication when the collateral duties of the interventions overwhelmed them. Convergent: “I think the benefits are for the Veteran, that they are getting their needs met more efficiently …” (Clinician 12) Divergent: “What was tricky about it was I had to catch the patient before they were discharged and since I didn’t have anyone on the inpatient side to tell me really when the patient was going home, like we looked at, OK, can we go to discharge planning rounds, but those were long. Those were like hours and hours, and so, again, I’m doing my other role, my full-time job, and then to attend discharge rounds, that wasn’t really that helpful.” (Clinician 20)
Accurate Communication	Increase: 33.47% (*n* = 6) Decrease: 3.52% (*n* = 3)	Accurate communication has one of the largest discrepancies in scores, with one-third of Veterans Health Administration (VA) sites experiencing a decrease. A lack of knowledge concerning the interventions could have impacted this. Convergent: “So, I mean, I think that the best part of the team, and I think this is something that has been, that has come up a lot on the team is that it works, it’s a very tightly knit team. There’s a lot of really successful collaboration. I think it’s a true multidisciplinary practice, you know, where, you know, rather than all the specialties kind of working in parallel, everybody really synchronizes and sort of provides their expertise in a real multimodal way, and there is just, it’s just a very collegial environment, you know, with really excellent collaboration.” (Clinician 23) Divergent: “If people would just communicate. It would be so much easier. And I think that that’s what the concept of [the intervention] is. It’s improving our communications across the board, discussing – making sure the patient has what they need before they leave. And then they have to – so when they leave and they don’t have that, they’re calling the system. Hard to explain what the … role is.” (Clinician 57)
Problem-Solving Communication	Increase: 7.19% (*n* = 8) Decrease: 6.8% (*n* = 1)	Problem-solving communication increased due to staff dedication to successfully implement and sustain the intervention despite facing numerous challenges. Convergent: “I would say helping Veterans out, identifying problems before they turn into bigger issues, and being part of a process to fix problems.” (Clinician 81) Divergent: “It’s always frustrating to identify a problem that you can’t solve. … I think a lot of times in the VA, if some, if there’s an issue and it’s not something that you’re solely responsible for, you just pass the buck, and you don’t necessarily make sure it’s to the right person who can fix the problem because it’s not technically your responsibility.” (Clinician 17)
Shared Goals	Increase: 12.85% (*n* = 6) Decrease: 2.55% (*n* = 3)	Shared goals increased for two-thirds of the sites. When shared goals decreased there was a lack of unified purpose and understanding for the interventions. Convergent: “… everyone on this team really gave 100 percent every time, you know, really working because we really believe in what we do. We believe in ourselves. We’re very – we’re all very strong personality, so we make a dynamic team. Yeah. We support each other.” (Clinician 43) Divergent: "There is a lack of unified purpose and understanding of the intervention at the site level, despite the setting implementation goals from national, many front line staff were unclear on the bigger educational plan and the intervention process at their sites. I mean, we have a lot of these like implementation goals or what they call milestones to meet … and not necessarily a very clear understanding of what the purpose of that was always.” (Clinician 25)
Shared Knowledge	Increase: 12.82% (*n* = 8) Decrease: 6.25% (*n* = 1)	Shared knowledge increased at most VA sites, and it helped not only with intervention delivery, but also with staff morale and well-being. Convergent: “We started doing these annual retreats for our team to kind of meet together talk a little bit about like our professional work, but also focus a lot on our quality of life as humans. And they’ve done some presentations and demonstrations around that, which has been really cool … They led us through doing like a Whole Health inventory questionnaire and applying it to our lives so we could see the work that they’re doing with patients on a daily basis, but also, you know, use it to improve our lives. So I think just kind of integrating them in those ways has been really cool and helpful.” (Clinician 35) Divergent: “… my feedback was always I don’t really think they know who we are, like what we’re doing, what we’re asking them, what we’re helping them with …” (Clinician 57)
Mutual Respect	Increase: 8.17% (*n* = 7) Decrease: 1.51% (*n* = 1) No Change: 1 site	Mutual respect increased at most sites, cases where it did not were rare outliers in the qualitative data and reflected a lack of general buy in. Convergent: “And you have this group of peers from all different disciplines who are saying, yeah, you know, you’re right. And it’s validating. I think it’s really validating that you – you’re being – your work is being given credit and value, even when it feels like maybe you’re not necessarily reaching a Veteran. But someone else knows you’ve tried, I guess, because there’s a lot of stuff that we do every day that, you know, we’re not going to email our supervisor a synopsis of our day every day.” (Clinician 85) Divergent: “And there was some resistance [integrating the intervention], from my perspective, it wasn’t a big deal, but yet it just wasn’t happening. And we’ve had some challenges when it comes to leadership. And I don’t think they understand exactly what we do or if they really care.” (Clinician 96)

*Note.* Abbreviations: Veterans Health Administration (VA)

### Relational facilitation impact

Overall, participants reported positive experiences with relational facilitation, though perceptions of specific activities varied by site. Many clinicians found the relational mapping activity particularly valuable for visualizing team roles and understanding the interconnectedness in the care coordination process:

*… before doing this and seeing, I didn’t realize how much we actually are in the middle and communicate and work with all the groups surrounding Veterans when they’re admitted … we kind of are just that middle person and communicate with all, everybody involved.* Clinician 49

Relational maps also helped identify overlooked roles and improve communication pathways:

*I think maybe just seeing visually … who all is involved in the team and just refreshing yourself on that, like we don’t forget, we can reach out to speech therapy … they’re just looking at the team as a whole for resources.* Clinician 7

*… I think that exercise was really good because it identified the strengths and weakness of the connection to … the different roles, and it identified where issues lie with how I needed to communicate better.* Clinician 6

While some participants did not recall the relational mapping activity, many described the SMART goal-setting process as a useful tool for engaging staff and driving participation. Additional insights into the impact of relational facilitation are summarized in Additional file 4.

## Discussion

Relational facilitation was successfully implemented across 15 geographically diverse VA sites. Improvements were observed in most RC Index scores and most RC dimensions, particularly in accurate communication and shared knowledge. Qualitative data revealed that relational facilitation fostered team resilience, collaboration, and innovation despite systemic challenges. To the QA-QUERI team’s knowledge, few studies in the literature report mixed methods data on RC and even fewer examine changes in RC over time [14, 34–36]. This manuscript addresses that gap by presenting a longitudinal, multi-site evaluation of RC within and between clinical teams, using both quantitative and qualitative data. It also introduces and details the relational facilitation implementation strategy, which was well received by staff and showed promising inclinations in improving RC. The relational facilitation step-by-step process is outlined in the Relational Facilitation Guidebook, offering a replicable process for other teams.

The relational mapping and SMART goal setting were perceived as useful, helping teams visualize roles, identify communication gaps, and engage in targeted improvement efforts. While quantitative data showed modest gains in problem-solving communication, the qualitative narratives highlighted both the strength and strain of clinicians’ adaptive efforts under resource constraints. The support provided by the QA-QUERI facilitation team was a benefit to many of the participants and supports the science and practice of facilitation to support teams implementing evidence-based interventions [21]. Accurate communication was the most improved dimension, yet also the most context-sensitive, with some sites reporting declines due to organizational uncertainty and policy changes. As the VA continues to experience significant changes [37, 38], clinical teams would benefit from relational facilitation support to ensure workforce stability and efficiency during implementation of new interventions.

Across the sites, RC Index scores from pre- to post-RC Surveys increased at seven sites and decreased at two. The decline at one site (VA 2) may be attributed to a broader rollout of the intervention and a significantly larger post-survey sample, potentially diluting the perceived impact. At another site (VA 14) substantial staff turnover and a national hiring freeze led to increased collateral duties for remaining staff, likely contributing to the decline in RC scores. These findings align with existing literature that describe how RC can drive change in high functioning environments [14, 39]; however, it is more difficult to engage staff effectively in change efforts in settings burdened by resource constraints [22].

QA-QUERI implementation coincided with major national disruptions, including the COVID-19 pandemic, multiple VA hiring freezes, and political uncertainty [37, 40], all which led to unprecedented levels of burnout and stress across healthcare settings [41, 42]. These factors created a climate of stress, high burnout, and job insecurity, which could have reasonably led to declines in RC [40, 41, 43]. Maintaining stable RC scores under such conditions would have been notable; yet, seven sites saw improvements. This contradicts literature which suggests that uncertain environments erode team dynamics and instead supports evidence that RC components can foster resilience and facilitate change even in challenging contexts [6, 22, 34, 42].

Although this evaluation lacked a control group and cannot establish causality, the observed improvements in the RC Index and dimension scores suggest an optimistic association with relational facilitation activities. The rate of improvement exceeded expectations given the implementation context. The structured support, tools, and guidance were successfully implemented and participants reported that the method “made sense”, clarified roles, and provided direction for implementing the QA-QUERI care coordination interventions. The favorable reception of relational facilitation underscores the potential utility of this method in complex healthcare environments.

### Strengths and limitations

Strengths of relational facilitation include its structured approach, clarity of goals, and ease of implementation. The Relational Facilitation Guidebook provided a clear roadmap, enabling QA-QUERI facilitators to implement the strategy effectively. Grounded in RC theory and implementation facilitation, relational facilitation enhanced team communication, clarified roles, and supported productive collaboration across sites. Limitations included lower post-SMART goal implementation response rates, independent samples, and the absence of a control group, limiting statistical inference and generalizability. Differences in site engagement, staffing, and the timing of the intervention wrap-up affected implementation fidelity and outcomes. Lastly, external pressures, such as the COVID-19 pandemic, hiring freezes, staff turnover, and political instability influenced both implementation and evaluation processes, and staff perceptions.

## Conclusion

This longitudinal, multi-site evaluation demonstrates that relational facilitation is a promising implementation strategy for enhancing RC among clinical teams, even in the face of significant organizational and environmental changes. The Relational Facilitation Guidebook offers a practical resource for teams seeking to improve care coordination and team functioning. Further research should explore the scalability of relational facilitation in other healthcare systems and examine its long-term impact on team performance and patient outcomes. This study contributes to the growing body of literature on RC and implementation facilitation by offering a replicable model for implementation and highlighting the resilience of clinical teams when equipped with the right tools and support.

## Electronic supplementary material

Below is the link to the electronic supplementary material.

Supplementary Material 1

Supplementary Material 2

Supplementary Material 3

Supplementary Material 4

## Data Availability

Relevant data not already included in this article is available upon request via the corresponding author.

## Abbreviations

CCICM: Care Coordination and Integrated Case Management
MISSION: Maintaining Internal Systems and Strengthening Integrated Outside Networks
QA-QUERI: Quadruple Aim Quality Enhancement Research Initiative
QUERI: Quality Enhancement Research Initiative
RC: Relational Coordination
SD: Standard Deviation
SMART: Specific, Measurable, Attainable, Relevant, Time-based
TNP-HHC: Transitions Nurse Program with Home Health Care
VA: Veterans Health Administration
WHCPMT: Whole Health Coaches in Pain Management Teams
