# Supplementary material for: Enhancing veteran care through relational facilitation: strategies and mixed methods outcomes
Source: Implement Sci Commun. 2026 Aug 6;7:221. doi: 10.1186/s43058-026-01065-z (PMC13737519; doi:10.1186/s43058-026-01065-z)
Supplement: Supplementary file 2 [file 43058_2026_1065_MOESM2_ESM.pdf]

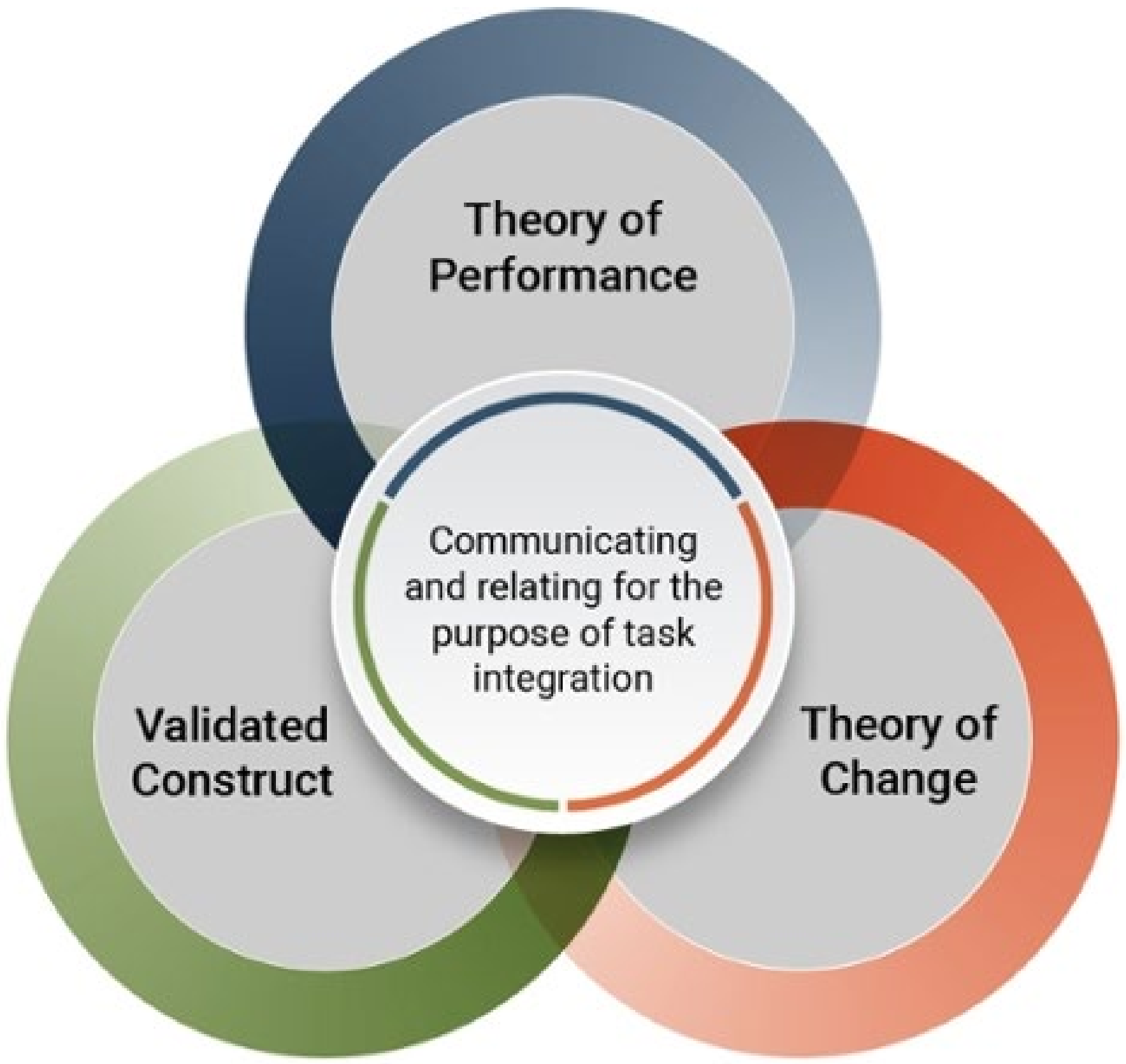

RELATIONAL COORDINATION
RESEARCH COLLABORATIVE

VETERANS HEALTH ADMINISTRATION NAME

Relational Coordination Progress Report

PRESENTER NAMES

PRESENTER AFFILIATION

DATE

Pre-SMART Goal Relational Coordination Survey Results

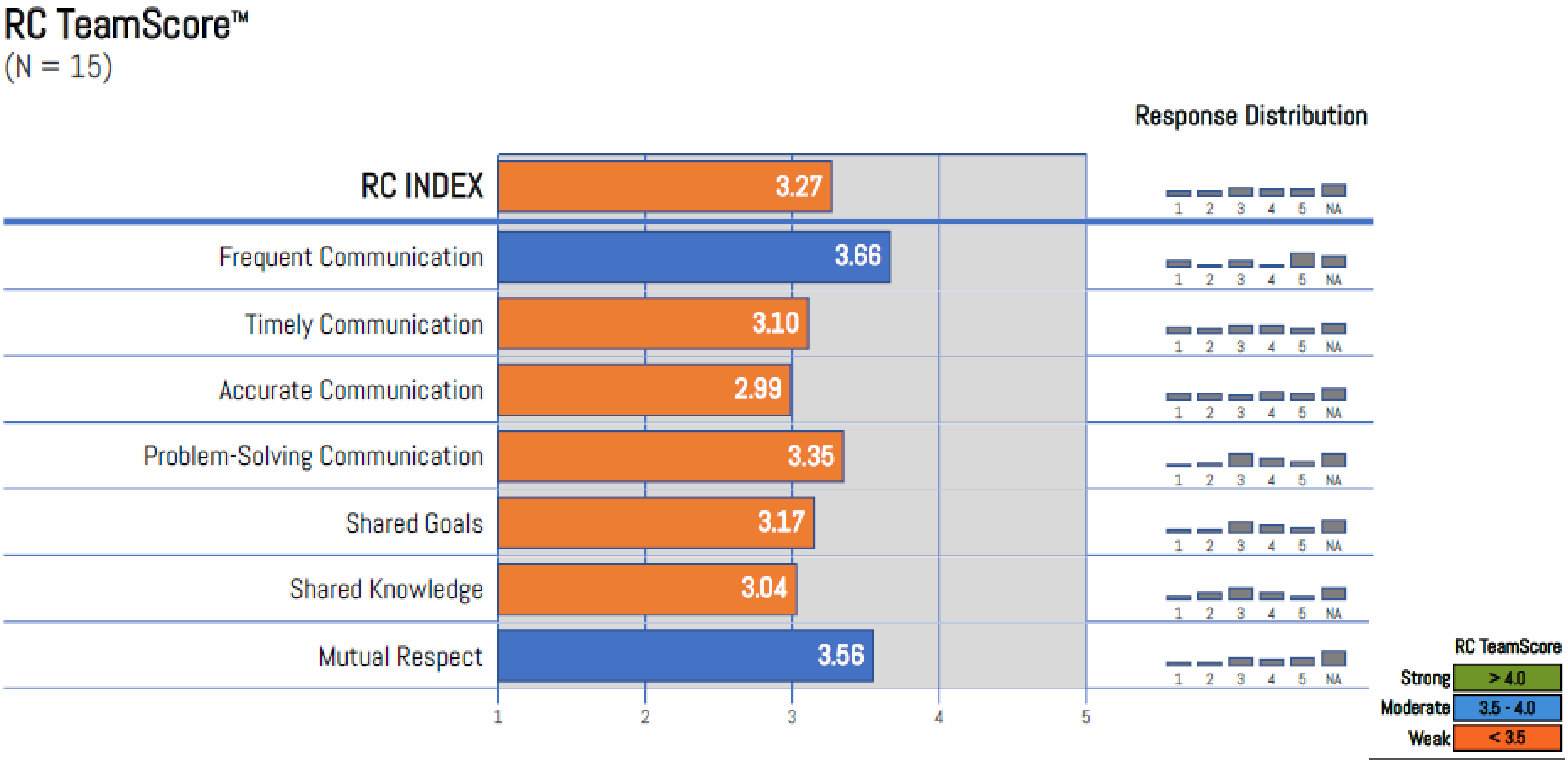

SMART Goal

Recruit and train moderators from nursing and social work by May 4, 2023

Relational Coordination Progress Report DATE

VETERANS HEALTH ADMINISTRATION NAME

Relational Coordination Survey Response Rates

PRE-SURVEY DATE v. POST-SURVEY DATE

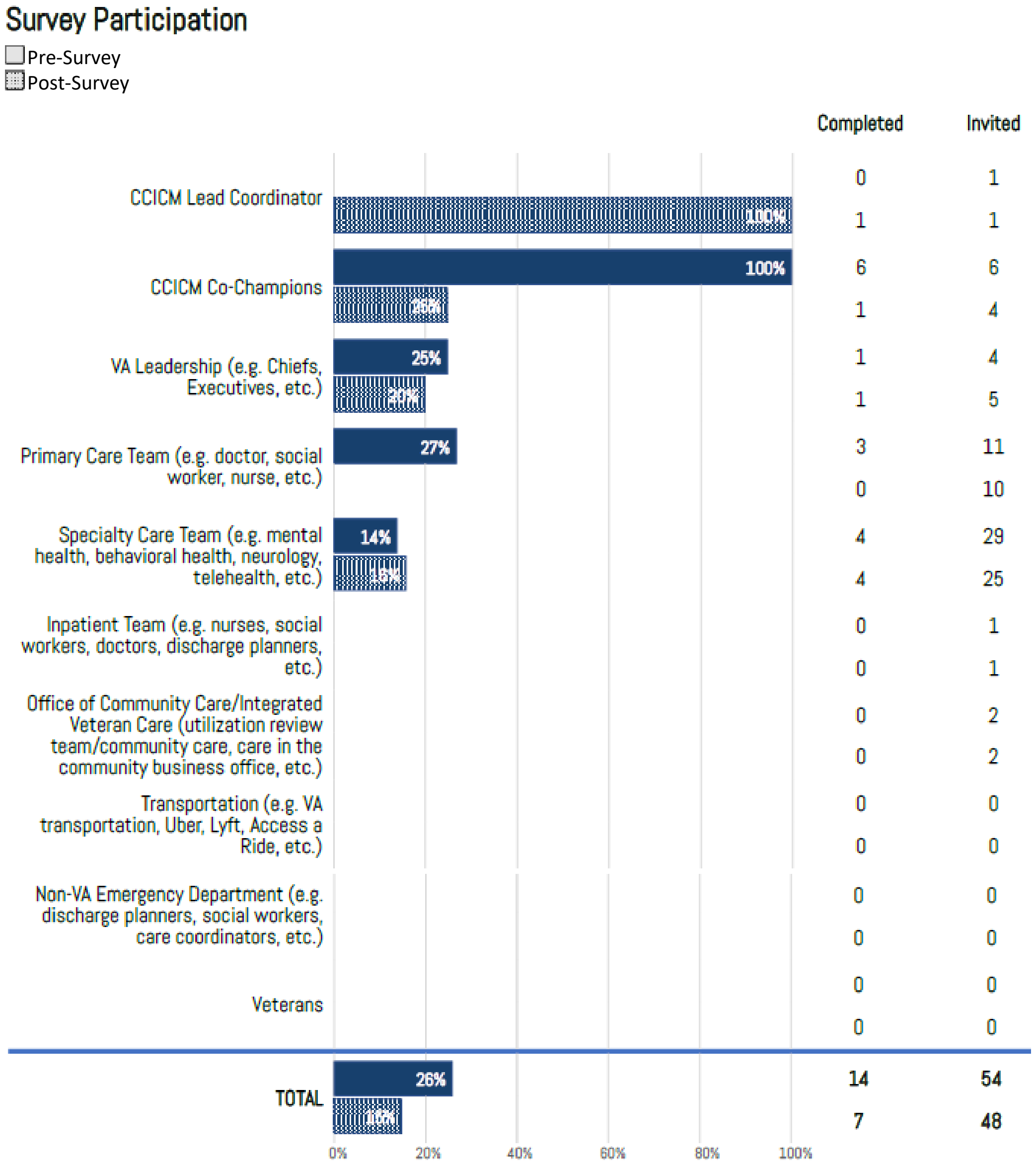

VETERANS HEALTH ADMINISTRATION NAME

Relational Coordination Progress

PRE-SURVEY DATE v. POST-SURVEY DATE

	RC TeamScore
Strong	> 4.0
Moderate	3.5 - 4.0
Weak	< 3.5

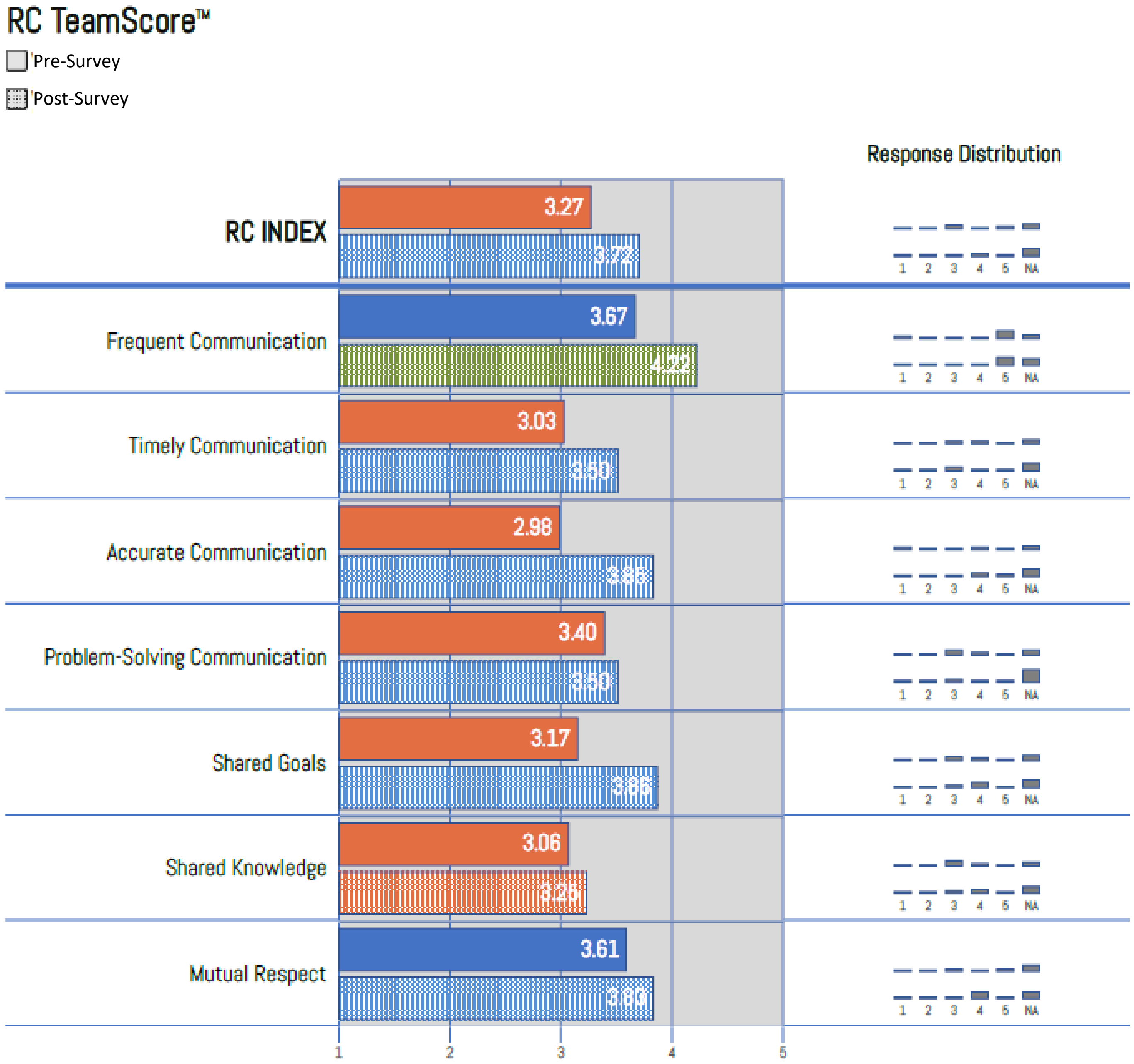

Average Ties – Relational Maps Comparison

Pre-Survey Relational Map

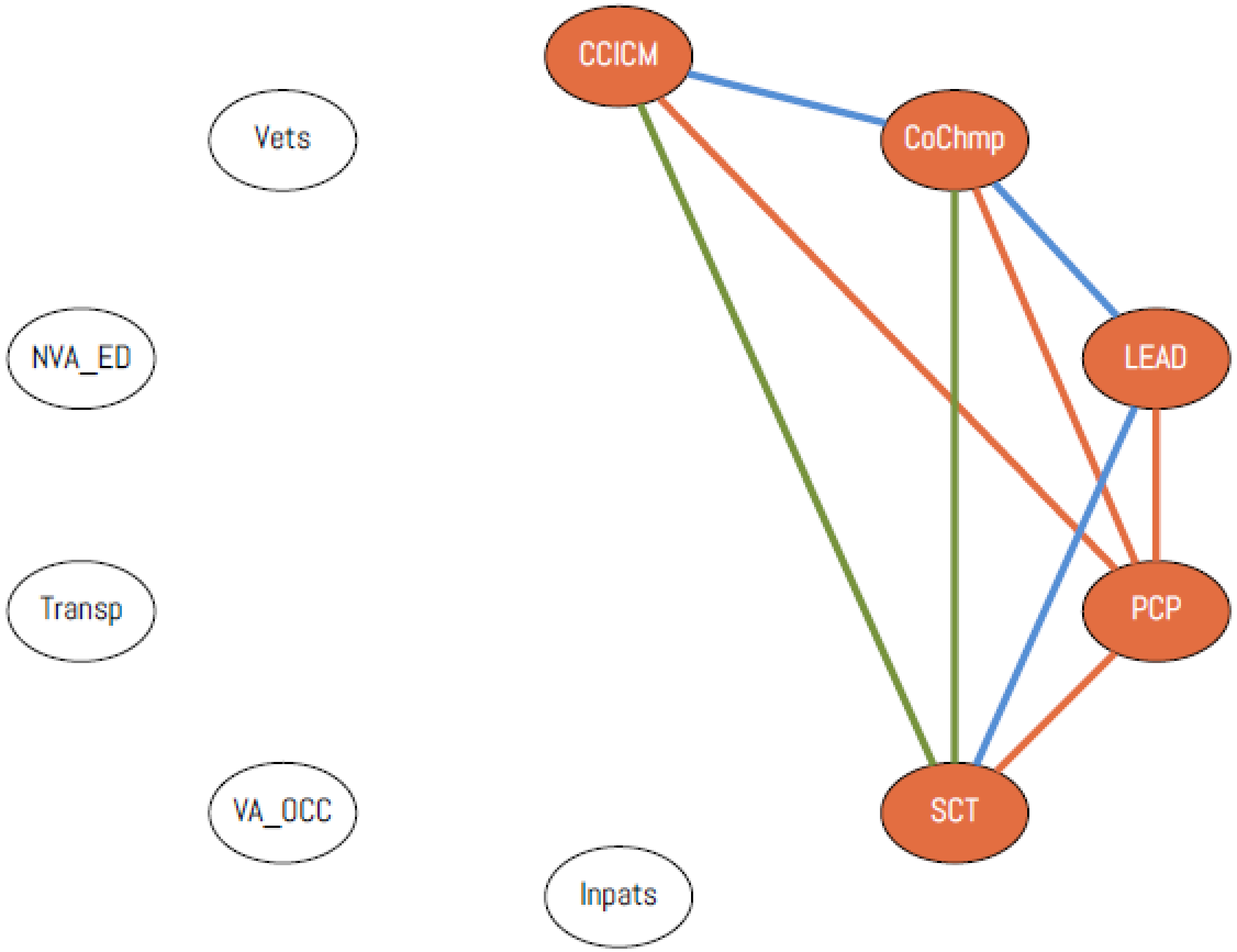

Post-Survey Relational Map

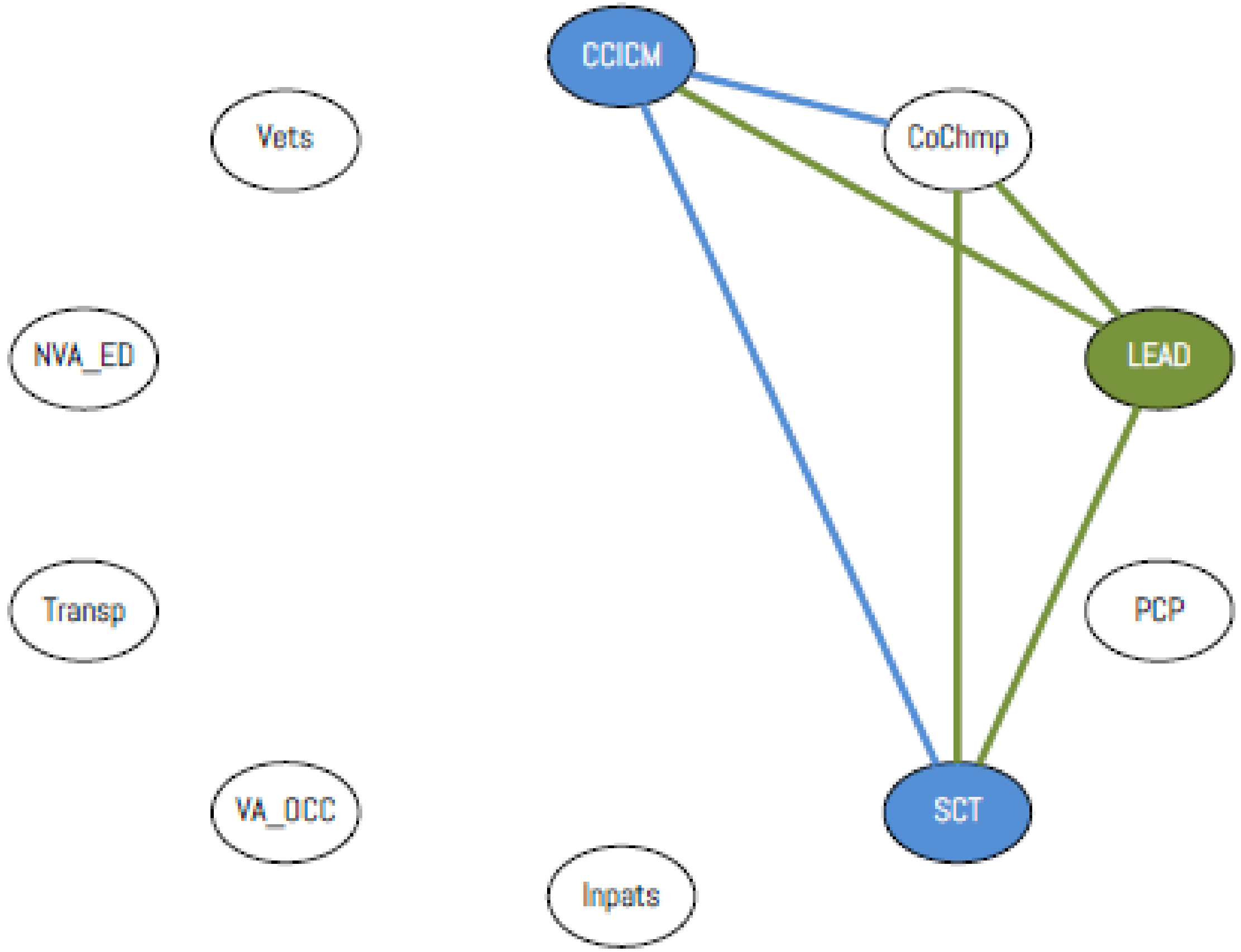

	Between	Within
Strong	[illegible]	[illegible]
Moderate	[illegible]	[illegible]
Weak	[illegible]	[illegible]

Reflections

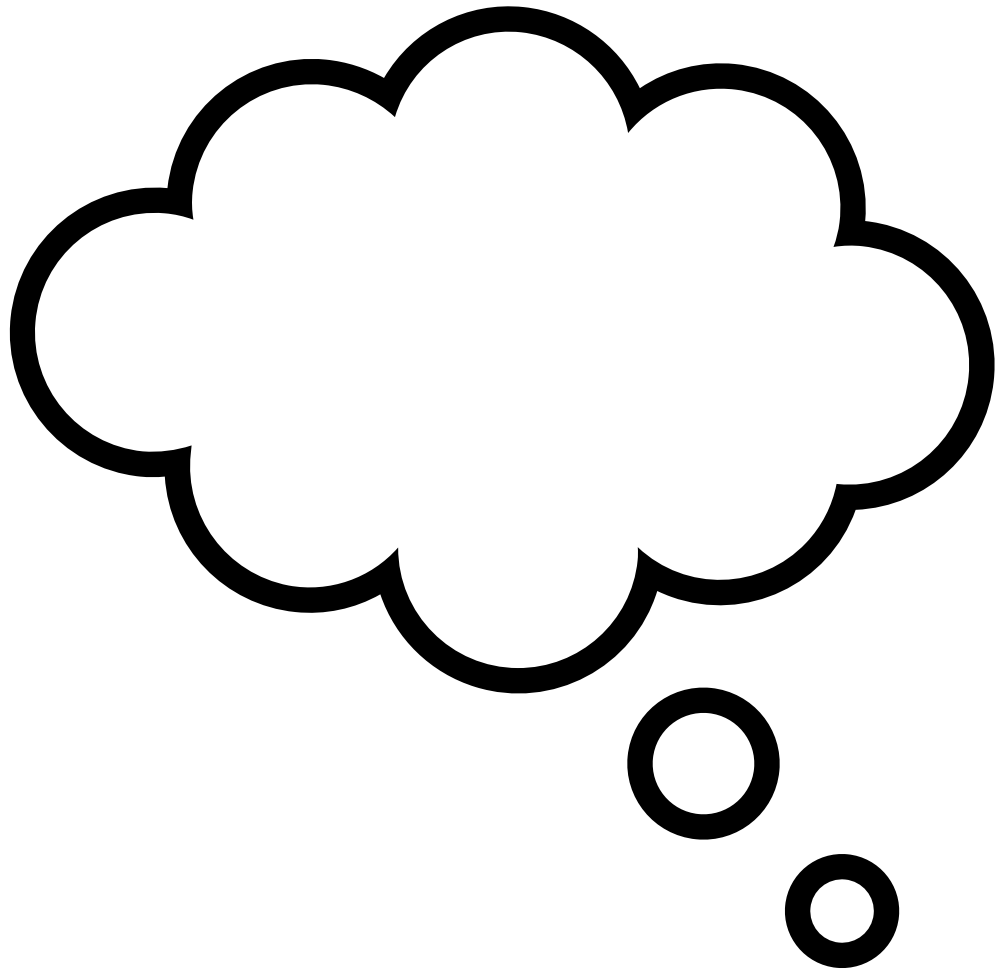

- Reflection on results
- What was your experience of focusing on relationships and communication within and between teams?
- Have you experienced any benefits (efficiency, quality, ease of work, well-being) from these efforts?
- How can you keep this progress going?
