# Supplementary material for: Enhancing veteran care through relational facilitation: strategies and mixed methods outcomes
Source: Implement Sci Commun. 2026 Aug 6;7:221. doi: 10.1186/s43058-026-01065-z (PMC13737519; doi:10.1186/s43058-026-01065-z)
Supplement: Supplementary file 3 [file 43058_2026_1065_MOESM3_ESM.docx]

Hello **[Mr./Ms. interview participant name]**,

My name is [interviewer name] and I am part of a quality improvement team looking at the implementation of Whole Health coaches in pain management teams. You may remember being interviewed previously by the QUERI team. We now would like to conduct a final follow up interview to see how Whole Health implementation is progressing. Findings from these interviews will support implementation of Whole Health across multiple sites.

We will not identify you as a participant in our report. Your participation is completely voluntary. You can stop the interview at any time and let us know if you’d rather not answer a particular question. Do you have any questions?

In order to make sure we capture all of the information you give us, we would like to record this call. The audio-file for the recording will be uploaded to a restricted access file on the VA intranet immediately after we complete this interview and will not have any identifying information, such as your name on the recording. Is this okay with you? If yes, I will ask you this question again once the audio recording begins.

We will now begin the audio recording: To make sure I have it on record,

1. Do you give us permission to record this interview?
2. Do you give us permission to contact you at a later time for a follow-up interview?
3. If we did have additional questions from the information you provide us today, can we contact you about it?

***Grounded prompts: If responses are limited or require clarification, probes may be used to elicit more detailed responses. Probes should use words or phrases presented by the participant using one of the following formats:***

***1. What do you mean by ____________.***

***2. Tell me more about ____________.***

***3. Give me an example of ____________.***

***4. Tell me about a time when ___________.***

1. Tell us about your experience working in pain management teams?
   i. Tell me about the individuals or departments that you work with most often to facilitate Veteran care for pain management.
   ii. What’s working well? What’s not working well?
       i. To what extent does your team work in a collaborative manner when caring for patients? (Team Effectiveness: Collaboration; Cohesiveness)
   iii. Tell me about the goals of the pain management interdisciplinary team(s)? (Relational Coordination: Shared Goals)
        i. What are your team’s shared goals?
        ii. How closely aligned are your goals with the goals of the team? (Relational Coordination: Shared Goals and Purpose)
   iv. To what extent do you feel that you have a well-defined role on the team? (Team Effectiveness: Role clarity)
   v. How do others know what you can offer them? (Relational Coordination: Shared Knowledge)
   vi. If you can, Describe the roles and responsibilities of others in your pain management team? (Team Effectiveness: Role clarity)
   vii. **If needed:** Describe the role of the Whole Health coach on your team? (Team Effectiveness: Role clarity)
2. Does the pain management team respect or support the work you do for Veteran pain management? (Relational Coordination: Mutual Respect)
3. How do you communicate with your pain management teams? (Relational Coordination: Communication)
   i. What’s working well? What’s not working well?
   ii. When a problem comes up, how do you go about solving it with your team? (Relational Coordination; Team Effectiveness: Conflict Resolution)
       1. Probe: Can you walk us through a time of when a problem came up in your team and how it was solved?
4. What challenges has your team experienced when implementing Whole Health Coaching with/in pain management teams?

(PRISM: Program/Intervention-Organizational Perspective)

i. How did you and/or your site overcome these challenges? (Relational Coordination)

5. What went well when implementing Whole Health Coaching in your pain management teams?

(PRISM: Program/Intervention-Organizational Perspective)

i. Why?
ii. What resources did you have to implement Whole Health Coaching with/in pain management teams? (PRISM: Program/Intervention-Organizational Perspective:

Organizational Context and Implementation Infrastructure)

6. How has Whole Health coaching impacted patients at your facility? *(PRISM: Program/Intervention-Organizational Perspective*: Characteristics of Organizational recipients)
7. How do you define success with Whole Health coach integration in a pain management team?

## **Relational Facilitation**

Now I am going to ask you about working with the QUERI implementation team. I want to ask about: Relational Facilitation, which includes the Relational coordination education session at PMOP conferences, the relational mapping, the Relational Coordination Survey, the Whole Health Coaching Sessions [which included SMART goals, survey debriefs, and personalized coaching]

8. In your own words, how did relational facilitation impact implementation of Whole Health in your pain management team? If at all…

**Sustainment/ Implementation Processes**

Next, I would like to ask a few questions about your plans to sustain Whole Health at your site*.*

9. What are your plans for sustaining the integration of Whole Health coaches in your pain management team?
10. Does leadership support the team’s work to sustain Whole health coaches in PM teams?
11. What strategies do you plan to use or are currently using to sustain Whole Health coaches in your pain management team?

**Implementer and Population Characteristics**

12. What are some of the benefits and challenges your site might experience in delivering the Whole Health Coaching in Pain Management Team model over time?

**EBI Characteristics**

13. What is the long-term value having Whole Health Coaches in Pain Management Teams?

**Inner Organizational Context; Implementation and Sustainability Infrastructure**

14. How is the current infrastructure supporting sustainment of Whole Health at your site?
15. What do you wish you have to sustain Whole Health coach integration into PM teams moving forward?

## **Adaptations**

Next, we would like to ask you some questions about any changes to the Whole Health program at your site. We expect that changes are made to programs to make them better fit the characteristics and needs of the site and the patients who are being served.

16. What were the most important changes made to the Pain Management Teams to help Whole Health coaches integrate?
    i. Why was this change made?
    ii. What impact did each of these changes have?

a. Are you planning any additional changes in the future to integrate WH coaches into PM teams?

17. Is there anything else you’d like to tell me about working in a pain management team at your site?

Thank you for participating in this interview.
