# Supplementary material for: Enhancing veteran care through relational facilitation: strategies and mixed methods outcomes
Source: Implement Sci Commun. 2026 Aug 6;7:221. doi: 10.1186/s43058-026-01065-z (PMC13737519; doi:10.1186/s43058-026-01065-z)
Supplement: Supplementary file 4 [file 43058_2026_1065_MOESM4_ESM.docx]

*Positive Impacts of Relational Facilitation*

| Impact Category | Qualitative Data Summaries and Relevant Quotes |
| --- | --- |
| Insights About Relational Coordination Survey | Sites expanded to include additional multidisciplinary clinicians to offer insights on how to help Veterans. The results of the Relational Coordination survey led to the establishment of shared governance at the time. The survey helped identify targets for improvements in implementation processes:  “That was great to see how when implementing this process, how it affected relationships, not just amongst us in the staff, but amongst the whole team in how we could better improve upon our strategies as we work closely together to implement [our intervention]. I feel that…the feedback that we got from the surveys that were done, actually helped us to project out and put us at different targets.” (Clinician 65)  For some sites, survey responses were low, especially from the roles that would be helpful to hear from.  “I remember having a meeting and discussing that survey and then after this, it wasn't a lot of, the bad thing about that for me was it wasn't a lot of feedback from whom I thought would be the essential stakeholders…” (Clinician 13) |
|  | The Relational Coordination Survey helped staff understand the bigger picture of how roles are connected and can be part of the team: “It just showed me the whole circle of everyone, all the team members. And in looking at the results from that survey, it just gave me a better understanding of where everyone was at... So basically, it was just a good snapshot of what everyone was doing and where everyone was.” (Clinician 34) “I think that initial survey, the result of that made it more apparent the need to have clarification on roles. So, I feel like that survey in itself provided an opportunity to kind of expand ideally what the potential for <my role> would be.” (Clinician 47) |
|  |  |
| Specific, Measurable, Attainable, Relevant, and Time-based, (SMART) Goal Impact | The site teams wanted to specify goals to work on and relational facilitation activities supported them in refining these and following through with them:  “…because it was something that we had talked about in our group, and then when we came to <Quadruple Aim Quality Enhancement Research Initiative>, we said, well, that's something we're going to do. So that's like a goal. So it probably was not identified specifically as a SMART goal to the rest of the team.” (Clinician 53)  The SMART goal was aligned with the work taking place and is a good process to use to set goals:  “…it wasn’t a brand-new idea to me that was super helpful, but I do think in general, it’s a good way to approach making goals.” (Clinician 16)  “… it’s kind of breaking down and looking at the process step-by-step that we were already working on, so it wasn’t super helpful I think to meet in our group, but I think it’s a good concept anyway, and I think it can be helpful to some people who are struggling to identify problems and how to solve them.” (Clinician 76) |
|  |  |
| Other Insights and Impacts | Relational facilitation activities helped participants be more proactive in the workplace, build relationships, and helped with problem solving:  “I think just in general just learning to be focused on continuing to build relationships and ask questions and not being afraid to set up a meeting to get to know people and clarify what’s going on and how to direct patient care. I think it was just a good reminder that that’s a good thing to keep doing.” (Clinician 81)  “…relational coordination was a good reminder to not be afraid to ask questions and have meetings and make connections with people who impact the Veterans’ care and to be proactive about building those relationships and maintaining them.” (Clinician 64)  “…relational aspect - any time you run into an issue with a Veteran and care, make sure that you are handing it to the correct person and building relationships to the people who fix the, whatever problem it is, and over time, you will become more knowledgeable about how to approach issues and just problem-solving in general…” (Clinician 65)  Relational facilitation activities increased visibility for the intervention:  “…people come to us ‘cause they realize what we do now, it's good - we are that middle person. So, I feel like our communication has definitely increased and improved.” (Clinician 81) |
|  | As a result of relational facilitation activities, one intervention conducted team-building activities. Staff organized a half-day retreat focused on the intervention. The team did a personal health inventory and wrote personal mission statements, which improved team confidence about the intervention:  “I feel like everybody really leaned into the idea of whole health. It's just I think that the team now is able to speak more confidently to patients about the [intervention] experience, so it's not like our team values it more or differently because we valued it from the beginning. I think it's just more, other team members feeling more confident to talk to patients about it.” (Clinician 62)  Relational facilitation activities led to establishing a Veteran outreach letter:  “…that is what led to sending an outreach letter following a visit that Veterans have in the pain clinic. So that led to outreach. It gave us an idea for outreach.” (Clinician 45)  Relational facilitation activities led to developing an intervention orientation:  “Well, that, yeah, we kind of developed, that's when we developed our orientation actually so that we could invite other primary care providers or other specialty clinics or whatever to jump on the orientation so they could get an overview of [the] team.” (Clinician 86) |

*Note.* Abbreviations: Specific, Measurable, Attainable, Relevant, and Time-based (SMART)
